# Contribution of neural circuits tested by transcranial magnetic stimulation in corticomotor control of low back muscle: a systematic review

**DOI:** 10.3389/fnins.2023.1180816

**Published:** 2023-05-25

**Authors:** Mikaël Desmons, Michael Theberge, Catherine Mercier, Hugo Massé-Alarie

**Affiliations:** ^1^Center for Interdisciplinary Research in Rehabilitation and Social Integration (Cirris), CIUSSS de la Capitale-Nationale, Quebec, QC, Canada; ^2^Rehabilitation Department, Université Laval, Quebec, QC, Canada

**Keywords:** transcranial magnetic stimulation, low back muscle, corticospinal, paired pulse, neural circuits

## Abstract

**Introduction:**

Transcranial magnetic stimulation (TMS) is widely used to investigate central nervous system mechanisms underlying motor control. Despite thousands of TMS studies on neurophysiological underpinnings of corticomotor control, a large majority of studies have focused on distal muscles, and little is known about axial muscles (e.g., low back muscles). Yet, differences between corticomotor control of low back and distal muscles (e.g., gross vs. fine motor control) suggest differences in the neural circuits involved. This systematic review of the literature aims at detailing the organisation and neural circuitry underlying corticomotor control of low back muscles tested with TMS in healthy humans.

**Methods:**

The literature search was performed in four databases (CINAHL, Embase, Medline (Ovid) and Web of science) up to May 2022. Included studies had to use TMS in combination with EMG recording of paraspinal muscles (between T12 and L5) in healthy participants. Weighted average was used to synthesise quantitative study results.

**Results:**

Forty-four articles met the selection criteria. TMS studies of low back muscles provided consistent evidence of contralateral and ipsilateral motor evoked potentials (with longer ipsilateral latencies) as well as of short intracortical inhibition/facilitation. However, few or no studies using other paired pulse protocols were found (e.g., long intracortical inhibition, interhemispheric inhibition). In addition, no study explored the interaction between different cortical areas using dual TMS coil protocol (e.g., between primary motor cortex and supplementary motor area).

**Discussion:**

Corticomotor control of low back muscles are distinct from hand muscles. Our main findings suggest: (i) bilateral projections from each single primary motor cortex, for which contralateral and ipsilateral tracts are probably of different nature (contra: monosynaptic; ipsi: oligo/polysynaptic) and (ii) the presence of intracortical inhibitory and excitatory circuits in M1 influencing the excitability of the contralateral corticospinal cells projecting to low back muscles. Understanding of these mechanisms are important for improving the understanding of neuromuscular function of low back muscles and to improve the management of clinical populations (e.g., low back pain, stroke).

## 1. Introduction

Patients suffering different health conditions such stroke (Dickstein et al., 2004), spinal cord injury (Milosevic et al., 2015) and low back pain (MacDonald et al., 2009) present alterations in trunk motor control. These alterations are due to a lesion of the central nervous system (e.g., stroke and spinal cord injury) or reorganization in neural circuits (e.g., low back pain). For example, patients with low back pain exhibit differences in the excitability of neural circuits controlling trunk muscles (Massé-Alarie et al., 2012) and cortical reorganization of motor representation of trunk muscles in comparison to healthy participants (Tsao et al., 2011a; Schabrun et al., 2017). Nonetheless, testing neural circuits controlling trunk muscles in humans is challenging considering the anatomy of the trunk muscles (e.g., overlapping and adjacent layers of muscles, difficulty to stimulate nerves) and the organisation of the circuits within the central nervous system (e.g., small responses evoked by brain stimulation (Ferbert et al., 1992)). Thus, the neural control of trunk muscles in healthy humans remains little studied and our knowledge framework is often based on results from limb muscles in humans or from primate studies. Thus, improving our understanding of cortical organisation and neural circuitry involved in the control of trunk muscles in humans is critical.

Transcranial magnetic stimulation (TMS) is a non-invasive brain stimulation technique that has been extensively used to investigate corticomotor control. TMS activates corticocortical and corticospinal neurons and elicits a motor response recorded by electromyography (EMG) called motor-evoked potential (MEP). The MEP amplitude represents the excitability of the corticospinal projections to the targeted muscle (Reis et al., 2008). It encompasses excitability of both cortical elements depolarized by the induced current and of neural elements downstream on this pathway (e.g., spinal networks influencing α-motoneuron excitability) at the time of stimulation (Siebner et al., 2022). Numerous TMS protocols have been established to better understand underlying neural mechanisms involved in corticomotor control (e.g., pharmaco-TMS, epidural recording) (Ziemann, 2015; Di Lazzaro et al., 2018), but remain barely used to study the corticomotor control of proximal and axial muscles (e.g., low back muscles).

Results from functional, neurophysiological and TMS methodological studies suggest substantial differences in the role and the neural organisation of distal and axial muscles. For example, hand muscles are specialised in fine motor control such as writing or manipulation (Lemon, 2008) whereas low back muscles can be voluntarily activated to produce gross movement of the trunk or to control intervertebral motion (Bogduk, 2012). Low back muscles are also fundamental for postural and balance control (Massion, 1992). In general, the primary motor cortex (M1) has been suggested to be crucial for the execution movements of low back muscles (Ferbert et al., 1992; O’Connell et al., 2007). Evidence suggests that the supplementary motor area (SMA) is particularly important in postural control (e.g., anticipatory postural adjustment) (Massion, 1992; Jacobs et al., 2009). However, SMA has been also shown to be involved in cognitive processing (Nachev et al., 2008), movement preparation (Tanji, 2001), and voluntary movement control (Schramm et al., 2019). SMA may play similar roles in the control of low back muscles. Considering the execution of hand movements is controlled mostly by dense area of cortical neurons in M1 (Strick et al., 2021), the depolarization of axons within hand muscles M1 representation using TMS is easy (Groppa et al., 2012). In contrast, the activation of cortical neurons projecting to low back muscles is challenging (Ferbert et al., 1992). Indeed, some methodological factors need to be considered when low back muscles M1 representation is targeted by TMS. For example, it is often essential to stimulate at very high intensity (e.g., 100% of the stimulator output (O’Connell et al., 2007; Tsao et al., 2011a,b)) or to use of a powerful coil (e.g., double cone) (Nowicky et al., 2001; Strutton et al., 2005), and to require voluntary contraction (Ferbert et al., 1992) to increase corticospinal excitability and elicit MEPs. Despite these methodological adaptations, only MEPs of small amplitudes are obtained (Ferbert et al., 1992; Strutton et al., 2005). These specificities seem to correspond with the limited contribution of M1 in low back muscles control suggested by studies in primates (Lawrence and Kuypers, 1968a,b). Despite these differences, corticomotor control of low back and distal muscles also share similar mechanisms. Paired pulse TMS protocols (e.g., short intracortical inhibition (SICI), short intracortical facilitation (SICF)) applied on low back muscles M1 representation have been successful in reducing or increasing the size of the MEP, respectively (Massé-Alarie et al., 2016b; Chiou et al., 2020). Thus, it is suggested that low back muscles are controlled or organised distinctly from distal muscles in the central nervous system (e.g., different neural areas, different densities of cortical neurones) (Massion, 1992), but they could also depend on similar M1 circuits influencing the excitability of the corticospinal cells (Massé-Alarie et al., 2016b).

Although the number of TMS studies targeting low back muscles is limited compared to hand muscles, an increasing number of studies are published each year. A review of the literature will help to understand better this control in humans. The main objective of the present study was to systematically review the neural control of low back muscles in humans tested by TMS. To address the primary objective, three secondary objectives were pursued: (i) to measure the MEP latency of low back muscles, (ii) to measure the latency and duration of the silent period, and (iii) the measure the effects of paired pulses protocols (e.g., intracortical inhibition, facilitation). For each of these objectives, comparisons between different conditions were done when it informed on specific neural mechanisms.

## 2. Methods

### 2.1. Data source and search

This systematic review was performed in accordance with the Preferred Reporting Items for Systematic Reviews and Meta-Analyses (PRISMA) guidelines (Page et al., 2021) and was registered in PROSPERO (ID number: CRD42020187517). An electronic bibliographical search was conducted in four databases: CINHAL, Medline (OVID), Embase, and Web of Science, from the date of inception on May 19th, 2022. No restriction about the year of publication was applied. A combination of thesaurus terms and free-text terms related to two different concepts were used to identify relevant articles: the use of experimental neurophysiological techniques and low back muscles (full search strategies available in Supplementary material 1).

### 2.2. Eligibility criteria

#### 2.2.1. Inclusion criteria

Considering that the current search strategies identified 105 studies (see next section), we decided to divide the included studies in two groups: (i) studies using TMS that inform on cortical circuits and corticospinal projections to low back muscles and (ii) studies using stimulation of the peripheral nervous system (e.g., vestibular, cutaneous electrical stimulation) informing on spinal and subcortical control of low back muscles. This systematic review reports only results from included studies using TMS of low back muscles. This is a deviation from the published protocol. The eligibility criteria presented below was the one registered in Prospero regardless of the neurophysiological technique used.

Studies were included if:

- the studied participants were healthy adults;
- low back muscle activation was measured using electromyography (EMG recording of paraspinal muscles between T12 and L5 levels is considered as low back muscle activation);
- the use of a neurophysiological technique evoking a muscle response in lower back muscles (a neurophysiological technique is defined as an external device that activates elements of the nervous system) (peripheral or central) that may inform on neural circuits or pathways underlying the motor control of the lower back muscles (e.g., transcranial magnetic stimulation, vestibular electrical stimulation and muscle tap);
- studies that were written in English or in French.

#### 2.2.2. Exclusion criteria

Studies were excluded when they met the following exclusion criteria:

- studied subjects presented pain or any health conditions as the focus of the study (e.g., low back pain, scoliosis);
- low back muscle activation was not measured (e.g., studies using solely magnetic resonance imaging or electroencephalography);
- neurophysiological techniques were used but results did not inform on neural circuits (e.g., TMS-studies reporting only mapping parameters or amplitude of unconditioned MEP);
- mechanical perturbation (e.g., weight dropping) was not applied directly on trunk as it implies an indirect response of the lumbar muscles;
- studies realised on animals;
- the article was a conference abstract or was not original results.

### 2.3. Study selection

All records from databases identified by the literature search were imported on Covidence (Covidence systematic review software, Veritas Health Innovation, Melbourne, Australia). After removal of duplicates in Covidence, two independent reviewers (MD, MT) conducted the titles and abstracts screening based on the predetermined eligibility criteria. Potential articles that met the eligibility criteria were further reviewed (full-text screening) independently by two authors (MD, MT). A third reviewer (HMA) was involved to resolve any disagreement through open discussion and consensus at any steps of the systematic review.

### 2.4. Critical appraisal of studies

To assess the methodological quality of the included studies, the checklist proposed by Chipchase et al. (2012) was used (independent raters: MD, MT) (Supplementary material 2) (Chipchase et al., 2012). This checklist is a critical appraisal tool used to identify methodological factors that could influence the variability of TMS outcomes. Potential sources of methodological biases (30 factors) in the included articles were divided in 3 domains: participant (8 factors), methodological (20 factors) and analytical (2 factors). Factors are rated as either being either ‘reported’ and/or ‘controlled’ (Chipchase et al., 2012). To be considered as ‘controlled’, a factor could have been used as exclusion criteria (e.g., by excluding participants taking drugs acting on the central nervous system) or included in the statistical model as a covariate (Chipchase et al., 2012). In the current review, a factor was also considered as ‘controlled’ if it was sufficiently detailed to ensure that: (i) the methodology followed published guidelines [e.g., by determining the motor threshold based on validated procedures (Tranulis et al., 2006; Groppa et al., 2012)], (ii) procedures were strictly replicated throughout the study and across participants, and (iii) there was no obvious source of bias likely affecting reliability. Reviewers independently rated each included studies, compared their ratings and discussed disagreement before to reach a consensus. Gwet coefficients (AC1) were calculated between independent reviewers prior to the consensus to determine the inter-rater agreement for each criterion of each quality assessment scale (Wongpakaran et al., 2013). Benchmark scales for AC1 Gwet’s value, have been proposed (Wongpakaran et al., 2013): < 0 (poor), 0–0.2 (slight), 0.2–0.4 (fair), 0.4–0.6 (moderate), 0.6–0.8 (substantial), 0.8–1 (almost perfect) (Munoz and Bangdiwala, 1997).

### 2.5. Data extraction

Information about included studies was extracted by two authors (MD, MT): demographic characteristics of the studied samples (e.g., sex, age, height, hand dominance), study design and methods (e.g., stimulator brand, type of coil, pulse shape, orientation of the current induced, localisation and intensity of stimulation, hotspot determination, number of MEPs recorded, use of a neuronavigation system, identification of active/resting motor threshold, interstimulus interval), EMG methods (e.g., electrodes localisation, type of EMG recording, level of muscle activation and participant position at the moment of stimulation), TMS outcomes (e.g., MEP latencies, silent period duration, % MEP test [inhibitory/excitatory protocols]). Data only available in Figures was extracted using the PlotDigitizersoftware (version 4.2, Pacifica, California, United States) when necessary.

### 2.6. Data analysis

Weighted average was used to characterize TMS outcomes across included studies with similar experimental design. Weighted average represents an average of the measurement extracted from the studies while considering the number of participants in each included study (*n*). In addition, minimum and maximum was used to describe the dispersion of data.


weighetedaverage=sum(outcomes∗n)/sum(n)

The weighted average (min, max) is reported throughout the text unless otherwise specified.

Table 1 describes briefly the TMS terminology and outcomes discussed in this review.

**Table 1:** Definitions of TMS terminologies and outcomes discussed in the systematic review.

Variable	Definition
*TMS methodology*	
Vertex (Cz)	The vertex is the highest point of the head. It is located at the point Cz of the electrode nomenclature of the International Federation of Clinical Neurophysiology’s 10–20 system (Nuwer et al., 1998).
Hotspot	Coil optimal position on the scalp, at a given stimulation intensity, that elicits the largest MEP amplitude in the target muscle (Groppa et al., 2012).
Active or resting motor threshold (a/rMT)	The minimal intensity of stimulation needed to elicit a reliable MEP of minimal amplitude in the active or resting target muscle group (Groppa et al., 2012).
*Single pulse TMS*	
MEP onset latency	Time interval between TMS applied on the motor cortex and the onset of a MEP (Groppa et al., 2012).
Silent period duration (SP)	When stimulating the M1 representation of a preactivated muscle, the silent period is the time elapsing from the onset of the MEP to the resumption of sustained EMG activity (Groppa et al., 2012).
*Paired pulses TMS*	
Interstimulus interval (ISI)	Interstimulus interval between the two pulses.
Conditioning stimulus (CS)	Stimulation delivered prior to a test stimulus. The effects of the conditioning stimulus on the size of a test MEP depend on the stimulus intensity and the interstimulus interval.
Test stimulus (TS)	Test stimulus delivered after a conditioning stimulus. It evoked a MEP in the target muscle which reflect the effect of the conditioning stimulus.
Short intracortical inhibition (SICI)	Conditioned MEP obtained with a suprathreshold test TMS preceded by a subthreshold conditioning TMS (ISI: 1–5 ms) (Kujirai et al., 1993).
Long intracortical inhibition (LICI)	Conditioned MEP obtained with a suprathreshold test TMS preceded by a suprathreshold conditioning TMS (ISI: 100–200 ms) (Valls-Solé et al., 1992).
Interhemispheric inhibition (IHI)	Conditioned MEP obtained with a suprathreshold test TMS preceded by a suprathreshold conditioning TMS over M1 ipsilateral to the target muscle (ISI: 4–50 ms) (Ferbert et al., 1992).
Short or long afferent inhibition (S/LAI)	Conditioned MEP obtained with a suprathreshold test TMS preceded by a conditioning afferent stimulus (e.g., electrical nerve stimulation) (ISI: 20-50 ms (SAI) and 200-1,000 ms (LAI)) (Chen et al., 1999a; Tokimura et al., 2000).
Intracortical facilitation (ICF)	Conditioned MEP obtained with a suprathreshold test TMS preceded by a subthreshold conditioning TMS (ISI: 10–15 ms). (Kujirai et al., 1993)
Short intracortical facilitation (SICF)	Conditioned MEP obtained with a subthreshold test TMS preceded by a suprathreshold conditioning TMS at specific interval (ISI: 1.0–1.5, 2.5–3.0 or 4.5 ms) (Tokimura et al., 1996).

TMS, transcranial magnetic stimulation; MEP, motor-evoked potential.

Analyses were segregated using different combinations of methodological factors susceptible to influence the measured variables. For example, studies showed that AP-TMS current direction will elicit MEPs at shorter latency compared to PA-TMS (Desmons et al., 2021). In some cases, it was not possible to report the impact of these factors due to the lack of studies (e.g., no paired pulses TMS protocol was performed on the vertex using AP-TMS).

#### 2.6.1. MEP latency

*MEP latency* was analysed using different combinations of experimental factors that potentially impact on latency.

- *Stimulation location combined with side of EMG recording*: (vertex: left vs. right muscle; hotspot: contralateral vs. ipsilateral muscles). For this analysis, only PA-TMS current direction was considered since AP-TMS was available only in 2 studies (Fujiwara et al., 2009; Desmons et al., 2021). Other factors were pooled (e.g., spine level for EMG recording).
- *Current direction (*e.g.*, PA-TMS, AP-TMS)*: Considering that only one study tested AP-TMS on ipsilateral hotspot (Fujiwara et al., 2001) and that no study stimulated the vertex using AP-TMS, only studies that stimulated the hotspot and recorded contralateral MEPs were included in this analysis. Different spine levels were pooled.
- *Spine levels (Upper [T12-L2] or Lower [L3-L5] lumbar spine levels)*: Right and left MEP latencies evoked by vertex stimulation were averaged and pooled with contralateral MEP latency elicited by hotspot stimulation. We took this decision since vertex stimulation more likely elicits contralateral MEP from each M1 due to current spread and there was no difference between right and left MEPs (see Results). In addition, only PA-TMS was included in the analysis to limit the variability that AP-TMS MEP latency can introduce and since PA-TMS was investigated in most studies.

#### 2.6.2. Latency and duration of silent period

All studies were pooled together because of the heterogeneity of the experimental design and since no factor was identified as potentially influencing silent period variables.

#### 2.6.3. Paired-pulse protocols

*Paired-pulse protocols* analyses were segregated based on a combination of factors (descriptions of paired pulses protocols are available in Table 1):

- Type of protocol based on expected physiological effect (e.g., SICI [inhibition], ICF [facilitation], SICF [facilitation]) and current direction (PA-TMS vs. AP-TMS): Studies testing paired-pulse protocol always targeted the hotspot and recorded contralateral MEPs. Analyses were done separately for PA- and AP-TMS current considering studies observed larger inhibition using SICI (Desmons et al., 2021). Other factors were pooled.

## 3. Results

### 3.1. Characteristics of included studies

**Figure 1:**
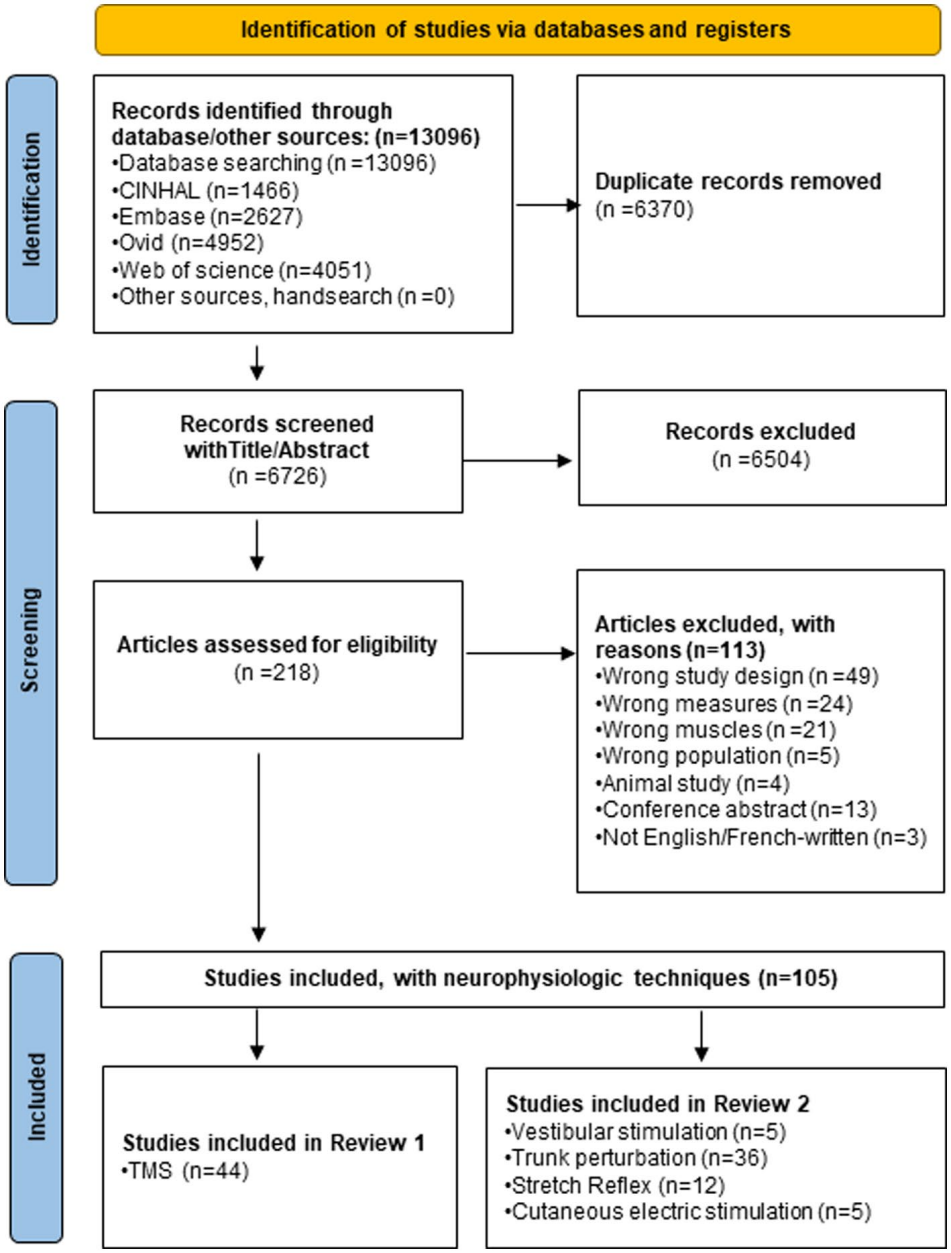
PRISMA flow chart of the systematic review.

**Figure 2:**
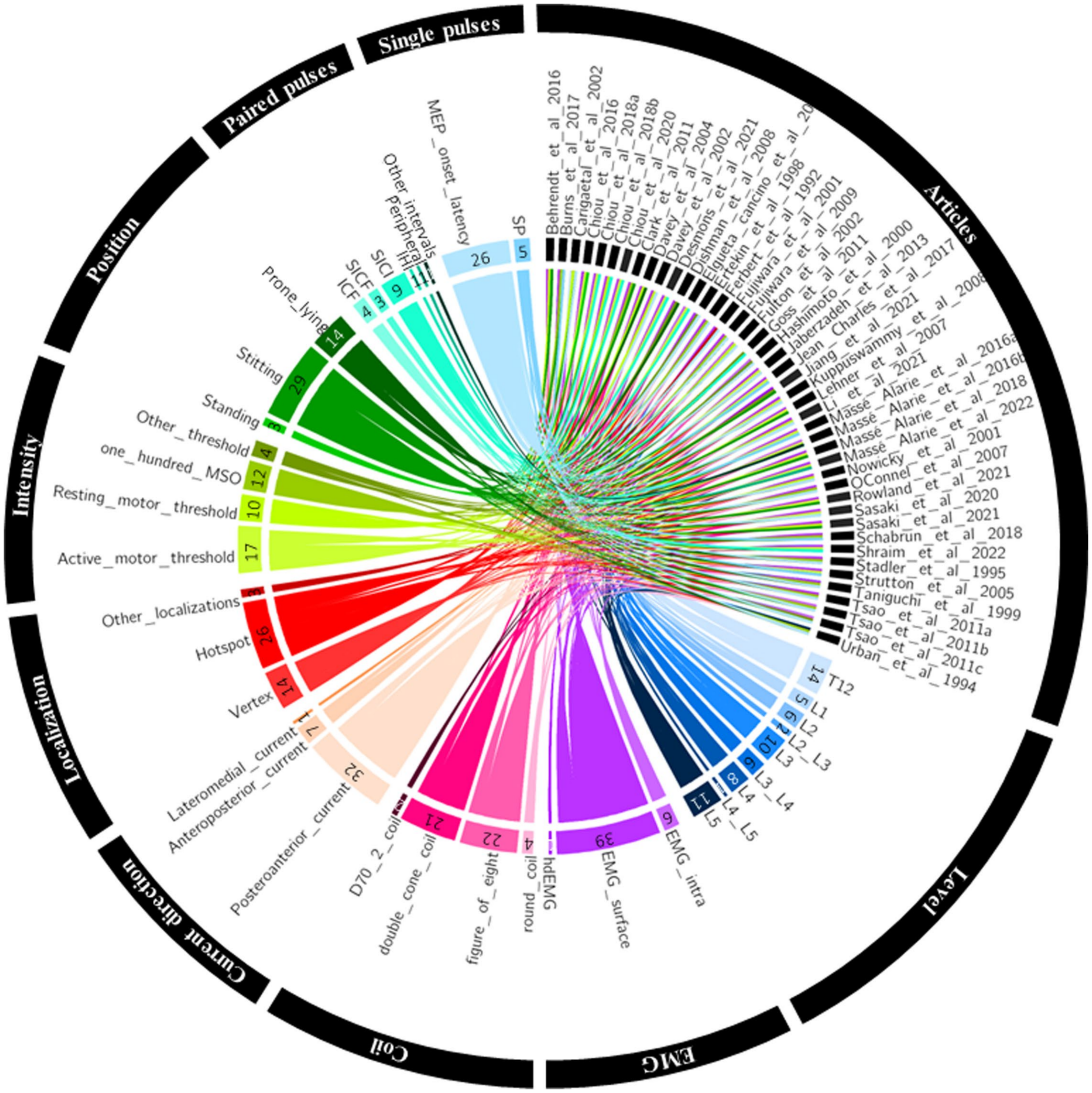
Circos plot linking the included studies to their methodological choices. EMG, electromyography; hdEMG, high density electromyography; IHI, interhemispheric inhibition; intra, intramuscular; ICF, intracortical facilitation; MEP, motor evoked potential; MSO, maximum stimulator output; SICF, short intracortical facilitation; SICI, short intracortical inhibition; SP, silent period.

Figure 1 summarised the results of the four-step systematic approach provided by the PRISMA guidelines: identification, screening, eligibility, and inclusion. From the 6,726 articles screened, 44 TMS studies targeting low back muscles were included in this systematic review (Figure 1). Figure 2 is a Circos plot giving a perspective of the multiple methodological choices used in the included studies and their combinations in each study. Outcomes describing the population of the included studies were described in Supplementary material 3 and TMS methodologies and Chipchase’s checklist scores are reported in Supplementary material 4. The mean sample size was 15.1 (3.0, 35.0) participants with a mean age of 27.8 (20.2, 48.1) years old.

### 3.2. Critical appraisal of the studies

Supplementary material 5 depicts for each study the number of ‘reported’ and ‘controlled’ factors of the Chipchase et al.’s TMS checklist. The median score was 60 (22.8, 96.8) % (Supplementary material 4). The *History of specific repetitive motor activity* (6.8% of studies) was the least reported factors. *Coil type*, *Coil orientation* and *Direction of induced current in the brain* were the least controlled factors (15.9%). AC1 of Gwet median coefficient was 0.9 (−1.0, 1.0) for reported factors and 0.9 (0.0, 1.0) for controlled factors both corresponding to an *‘almost perfect’* median interrater agreement (Supplementary material 6). Unfair agreement (corresponding to AC1 = −1) is explained by a misunderstanding of one item by one of the raters. A consensus was reach for each element of the checklist and resolved disagreements.

### 3.3. Methods of investigation

#### 3.3.1. TMS materials and setting

As shown in Figure 2, 27 studies investigated MEPs of low back muscles at the thoraco-lumbar level (i.e., between T12 and L2) and 36 between L3 and L5. Supplementary material 4 reports methodological details of included studies. Postero-anterior current in the brain was the most used (*n* = 32), followed by antero-posterior current (*n* = 7) and lateromedial current (*n* = 1). Five studies did not specify TMS current direction. TMS was mostly applied on a hotspot (*n* = 26) or on the vertex (*n* = 14). Three studies targeted multiple sites on M1. A motor threshold (MT) was used to define stimulation intensity in 27 studies (active motor threshold: *n* = 17 and resting motor threshold: *n* = 10). Eleven studies set the stimulation intensity at 100% of the maximal stimulator output (MSO) and six studies used a fixed arbitrary stimulation intensity of the MSO – usually without searching for MT. For example, 60, 80 and 100% MSO were used considering the difficulty to define a specific MT with high-density EMG (Jiang et al., 2021). One last study did not detail the procedure used to select TMS intensity (Goss et al., 2011). A double-cone and a figure-of-eight coil was used in 21 studies each. An alternative and more powerful version of the figure-of-eight coil, the D70^2^, was used in 2 studies. Finally, a round coil was used in 4 studies.

### 3.4. TMS outcomes

Twenty-nine studies reported latencies (MEP latencies: *n* = 26, corticospinal silent period latencies or duration: *n* = 5). Ten studies tested paired-pulse protocols (SICI: *n* = 8, intracortical facilitation (ICF): *n* = 4, SICF: *n* = 3, interhemispheric inhibition (IHI): *n* = 1, afferent conditioning: *n* = 1, other paired-pulse protocols: *n* = 1). Other paired-pulse protocols correspond to a conditioning stimulus (subthreshold) followed by a test stimulus (suprathreshold) at 6 to 9 ms ISI for which no consistent MEP modulation (inhibition/facilitation) of the MEPs have been observed yet (Massé-Alarie et al., 2016b).

#### 3.4.1. Single pulse TMS outcomes

##### 3.4.1.1. MEP latency

MEP latencies of all included studies are reported in Supplementary material 7.

- *Stimulation location combined with EMG recording side.* For vertex stimulation, six studies compared MEP latencies obtained from both the left and right back muscles using PA-TMS (left: 15.3 (13.5, 18.5) ms; right:15.3 (13.2, 19.5) ms) (Urban and Vogt, 1994; Nowicky et al., 2001; Cariga et al., 2002; Davey et al., 2002; Fulton et al., 2002; Kuppuswamy et al., 2008). For hotspot stimulation, one study measured latencies of the contralateral and the ipsilateral MEP (ipsilateral: 24.2 ms; contralateral: 19.5 ms) using PA-TMS (O’Connell et al., 2007). Five authors observed ipsilateral MEP when targeting the hotspot but did not report the MEP latency (Ferbert et al., 1992; Tsao et al., 2011a; Lehner et al., 2017; Jiang et al., 2021) or did not compare to contralateral MEP (Jean-Charles et al., 2017). One study reported contralateral and ipsilateral MEPs using AP-TMS from an average of multiple points over M1 (ipsilateral: 16.1 ms; contralateral: 15.7 ms) (Fujiwara et al., 2001).
- *Current direction.* Nine studies used PA-TMS, 2 AP-TMS (including one that tested both current directions) and 1 used latero-medial current direction (LM-TMS). The average latency was 15.0 (12.6, 19.5) ms with PA-TMS (Ertekin et al., 1998; O’Connell et al., 2007; Tsao et al., 2011b,c; Chiou et al., 2018a,b, 2020; Desmons et al., 2021; Li et al., 2021), 14.1 (13.1, 14.8) ms with AP-TMS (Fujiwara et al., 2009; Desmons et al., 2021) and 15.4 ms with LM-TMS (Jaberzadeh et al., 2013). In the only study that tested both PA- and AP-TMS (Desmons et al., 2021), the latency was significantly longer for AP-TMS (PA: 13.9 ms; AP: 14.8 ms – *p* = 0.017).
- *Spine levels.* Nine studies recorded MEP at the upper lumbar level (T12-L2) and 10 at the lower lumbar level (L3-L5). The average latency at T12-L2 was 15.5 (12.6–18.5) ms (Ertekin et al., 1998; Taniguchi and Tani, 1999; Cariga et al., 2002; Davey et al., 2002; Tsao et al., 2011b,c; Chiou et al., 2018a,b, 2020) and 16.2 (13.9, 19.5) ms at L3-L5 (Stalder et al., 1995; Ertekin et al., 1998; Nowicky et al., 2001; Fulton et al., 2002; Strutton et al., 2005; O’Connell et al., 2007; Kuppuswamy et al., 2008; Tsao et al., 2011b; Desmons et al., 2021; Li et al., 2021). Taniguchi & Tani observed a longer MEP latency at lower (19.5 ms) than upper (18.5 ms) lumbar levels (Taniguchi and Tani, 1999).

##### 3.4.1.2. Silent Period

MEP silent periods (SP) outcomes are reported in the Supplementary material 7. Contralateral SP variables were reported in four studies; all used PA-TMS current direction. Two studies reported a SP duration resulting in an average of 60.0 (38.5, 81.4) ms (Strutton et al., 2005; Massé-Alarie et al., 2016a) and two reported SP latency (45.1 (41.8, 47.8) ms) (Strutton et al., 2005; Burns et al., 2017).

#### 3.4.2. Paired-pulse TMS protocols

Supplementary material 8 presents results of paired-pulses TMS protocols which were tested in eleven studies. A wide range of conditioning stimulus, test stimulus and interval inter stimulus have been tested across studies. Except one study (Goss et al., 2011) (which used a fixed intensity instead of defining the intensity of stimulation relative to a motor threshold), all targeted the hotspot, recorded MEP on the muscle contralateral to the site of stimulation and used a motor threshold to define the intensity of stimulus. Definitions of paired pulses protocols are available in Table 1.

##### 3.4.2.1. Short-interval intracortical inhibition (SICI)

From the nine studies using SICI protocol, seven tested PA-TMS (only four reported results) (Massé-Alarie et al., 2016a; Chiou et al., 2018a,b, 2020; Desmons et al., 2021; Shraim et al., 2022) and four tested AP-TMS (including one that tested both current directions) (Goss et al., 2011; Massé-Alarie et al., 2016b; Elgueta-Cancino et al., 2019; Desmons et al., 2021). The weighted average conditioned MEP was 71.6 (52.9, 90.6)% of the MEP test with PA-TMS and 68.3 (64.8, 70.6)% of MEP test with AP-TMS. Only one directly compared PA- and AP-TMS and reported more inhibition with AP- than PA-TMS (PA-TMS: 90.6 (27.5)% of MEP test; AP-TMS: 64.8 (31.3)% of MEP test - p: 0.010) (Desmons et al., 2021). One study reported the percentage of participant with significant SICI (88% of participants) (Shraim et al., 2022).

##### 3.4.2.2. Intracortical facilitation (ICF)

Five studies tested ICF protocol, two used PA-TMS (only one reported data) (Desmons et al., 2021; Shraim et al., 2022), four used AP-TMS (including one that tested both current directions) (Goss et al., 2011; Massé-Alarie et al., 2016b; Elgueta-Cancino et al., 2019; Desmons et al., 2021). The conditioned MEP was 105.9% of MEP test with PA-TMS and 110.1 (90.6, 130.5)% of MEP test with AP-TMS. In the only study that compared PA- and AP-TMS, no difference in ICF was observed (PA-TMS: 105.8 (37.0); AP-TMS: 100.4 (35.6)% of MEP test; p: 0.909) (Desmons et al., 2021). One study reported ICF in 44% of participants (Shraim et al., 2022).

##### 3.4.2.3. Short intracortical facilitation (SICF)

Three studies tested SICF protocol, two used PA-TMS (Massé-Alarie et al., 2016a; Shraim et al., 2022) (only one reported data) and one used AP-TMS (Massé-Alarie et al., 2016b). The modulation was 195.5% of MEP test with PA-TMS and 161.3% of MEP test with AP-TMs. One study reported SICF in 44% of participants (Shraim et al., 2022).

##### 3.4.2.4. Interhemispheric inhibition (IHI)

One study tested inter hemispheric inhibition (IHI) (Jean-Charles et al., 2017) at ISI of 2, 4, 6, 8, 10, 12 and 40 ms. The only trend for a modulation of the MEP test was obtained at ISI 6 ms (76.4 (9.2)%), but was non-significant after correction for multiple comparisons.

##### 3.4.2.5. Afferent conditioning stimulation

One study tested afferent conditioning (also known as short/long afferent inhibition for TMS hand studies) (Massé-Alarie et al., 2022). Three peripheral stimulations were performed ((i) non-noxious and (ii) noxious (both with electrical current) and (iii) a muscle stimulation (with a figure of eight coil)) at ISIs of 20, 25, 30, 35, 40, 50, 60, 80, 100 and 200 ms prior to TMS. Only one condition elicited MEP modulation (−0.072 MEP/EMG ratio (log); ISIs = 60 ms; muscle stimulation) when accounting for EMG modulation.

##### 3.4.2.6. Other paired-pulse protocols

No modulation of MEP was present at ISI 5–9 ms using subthreshold CS and suprathreshold TS (Massé-Alarie et al., 2016b).

## 4. Discussion

The present systematic review provided an in-depth review of the quality and content of studies having tested corticomotor control of low back muscles with TMS in healthy adults. Although the heterogeneity in methodology in included studies is large, our review provided an overview of the potential organisation and function of corticomotor control of low back muscles. In the following sections, we will elaborate on: (i) the organisation of corticospinal projections to low back muscles; (ii) potential cortical circuits; and (iii) methodological aspects to consider in TMS studies targeting low back muscles.

### 4.1. Organisation of corticospinal projections to low back muscles

#### 4.1.1. Bilateral cortical projections to low back muscles

Contralateral MEPs to the targeted M1 are usually investigated, nonetheless ipsilateral MEPs of low back muscles have been frequently reported (Ferbert et al., 1992; Fujiwara et al., 2001; O’Connell et al., 2007; Tsao et al., 2011a,c; Jean-Charles et al., 2017; Lehner et al., 2017; Jiang et al., 2021). Two studies directly compared ipsilateral and contralateral MEP latencies (Fujiwara et al., 2001; O’Connell et al., 2007) which were similar in one study (Fujiwara et al., 2001) but different in a second one (ipsilateral MEP ≈ 4.7 ms longer than the contralateral MEP (O’Connell et al., 2007)). In the former, AP-TMS was used and latencies were an averaged from all sites of TMS including most medial sites which may have washed-out the differences due to depolarisation of neurons into the opposite M1 by current spread (Fujiwara et al., 2001). Similarly, when the vertex was targeted, no latency difference was reported between MEPs from left and right low back muscles (Urban and Vogt, 1994; Nowicky et al., 2001; Cariga et al., 2002; Davey et al., 2002; Fulton et al., 2002; Kuppuswamy et al., 2008), suggesting a depolarisation of neurons from both M1 (Tsao et al., 2008). Nevertheless, the difference in MEP latency between ipsi- and contralateral muscles (using stimulation at 100% MSO) observed in O’Connell et al., (2007) corresponds to results from hand studies (5–13 ms) (Ziemann et al., 1999; Chen et al., 2003; Strutton et al., 2004) and proximal and abdominal muscles (3–5 ms) (Tunstill et al., 2001; MacKinnon et al., 2004; Strutton et al., 2004; Tsao et al., 2008). The latency difference would be too long to be explained by current spread to the opposite M1 (Ziemann et al., 1999) and too short to result from transcallosal conduction (Chen et al., 2003). It is usually accepted that contralateral MEP originates from the activation of fast-conducting, monosynaptic cortico-motoneuronal neurons in M1 (Siebner et al., 2022). Similarly, the MEP latency approximatively corresponds to a conduction velocity that fits with the depolarisation of monosynaptic cortico-motoneuronal neurons (Ferbert et al., 1992). However, as reported in our review, large variability exists within and between studies in MEP latencies. Several authors points out the variability of the contralateral MEP latency of low back muscles up to ≈10 ms between participants (Ferbert et al., 1992; O’Connell et al., 2007; Desmons et al., 2021). This large variation cannot be fully explained by participants’ height (Taniguchi and Tani, 1999) and may question the fast-conducting and/or monosynaptic nature of the contralateral corticospinal projections to low back muscles in some participants with longer MEP latency. Large fibers dominate the descending volleys, but they constitute only ≈8% of the descending corticospinal fibers (Kraskov et al., 2019), hence, the proportion of smaller and slower corticospinal fibers depolarized could explain some of the variation observed. Further, disynaptic corticospinal pathways also exist (Strick et al., 2021) and may partially explain the longer latency. The variability between participants could reflect individual differences in the organisation of descending pathways projecting to low back muscles.

The longer ipsilateral MEP latency has been hypothesized to represent an oligosynaptic pathway (Ziemann et al., 1999; Chen et al., 2003) that may involve various combinations of synaptic connectivity between neural circuits and descending tracts at different levels of the central nervous system such pyramidal (e.g., ipsilateral uncrossed corticospinal tract (Ziemann et al., 1999; Chen et al., 2003; Strutton et al., 2004)), extrapyramidal (reticulospinal tract (Nathan et al., 1996; Ziemann et al., 1999)) and spinal circuits (interneuronal (Maxwell and Soteropoulos, 2020) and/or propriospinal circuits (Ziemann et al., 1999)). Indeed, there is evidence in humans and animals’ studies suggesting the presence of uncrossed ipsilateral corticospinal pathway by which an ipsilateral MEP may travel. Although most descending corticospinal fibers cross the midline at the pyramidal decussation, ≈20–30% do not cross (Kuypers and Brinkman, 1970; Nathan et al., 1990; Strutton et al., 2004) and originates from M1, premotor (PM) and supplementary motor areas (SMA) (Brinkman and Kuypers, 1973; Ralston and Ralston, 1985; Dum and Strick, 1991, 1996; Galea and Darian-Smith, 1994; Lacroix et al., 2004). In monkeys, more ipsilateral responses in trunk and upper limbs muscles were observed after stimulation of the SMA than M1 and PM (Montgomery et al., 2013). TMS studies in humans have also reported more occurrences of ipsilateral MEP in proximal and axial than distal muscles (Bawa et al., 2004; Strutton et al., 2004). It is suggested that bilateral corticospinal projections from a sole M1 may be advantageous for postural control that often necessitates bilateral activation of axial muscles (Tsao et al., 2008). However, there are many other neural structures that may – theoretically – contribute to the ipsilateral MEP. Considering the critical role of SMA in postural control and its high proportion of ipsilateral corticospinal fibers (Massion, 1992; Jacobs et al., 2009; Takakusaki, 2017), it is possible that ipsilateral MEP comes – at least partially – from SMA. Other authors suggest that TMS delivered over M1 can activate reticulospinal cells transsynaptically via corticoreticular connections (Fisher et al., 2012) since ipsilateral MEP latencies are modulated by rotating the head (Ziemann et al., 1999; Tazoe and Perez, 2014). Reticulospinal cells are known to be modulated by neck proprioceptors (Pompeiano et al., 1984), hence, ipsilateral MEP could represent a measure of the reticulospinal tract excitability (Maitland and Baker, 2021). Overall, although there is some evidence of corticospinal projections from a sole hemisphere bilaterally to low back muscles, the specific nature of descending pathways by which action potentials travel to low back muscles remain to be determined.

#### 4.1.2. Assessing various neural circuits by manipulating the TMS current direction

Manipulating the electrical current direction flowing in the brain elicited by TMS may probe different neural circuits. MEP latencies were mostly reported using PA-TMS (*n* = 32) whilst only one used AP-TMS (Fujiwara et al., 2009) and one compared PA- to AP-TMS (Desmons et al., 2021). Longer MEP latencies were observed with AP- than PA-TMS (Desmons et al., 2021) similarly to hand muscles (Sakai et al., 1997; Lazzaro et al., 2001). PA-TMS depolarises neural structures of the targeted cortical area (i.e., M1) (Siebner et al., 2022). It has been suggested that the longer MEP latency observed using AP-TMS could be the result of the depolarisation of axons of neurons from PM/SMA projecting to M1 (Volz et al., 2015; Siebner, 2020). Modeling of the electrical current flowing in the brain by TMS support these hypotheses (Aberra et al., 2020). Thus, future studies need to confirm if AP-TMS can specifically test circuits from SMA/PM while targeting low back muscles.

### 4.2. Neural cortical circuits involved in the control of low back muscles

**Figure 3:**
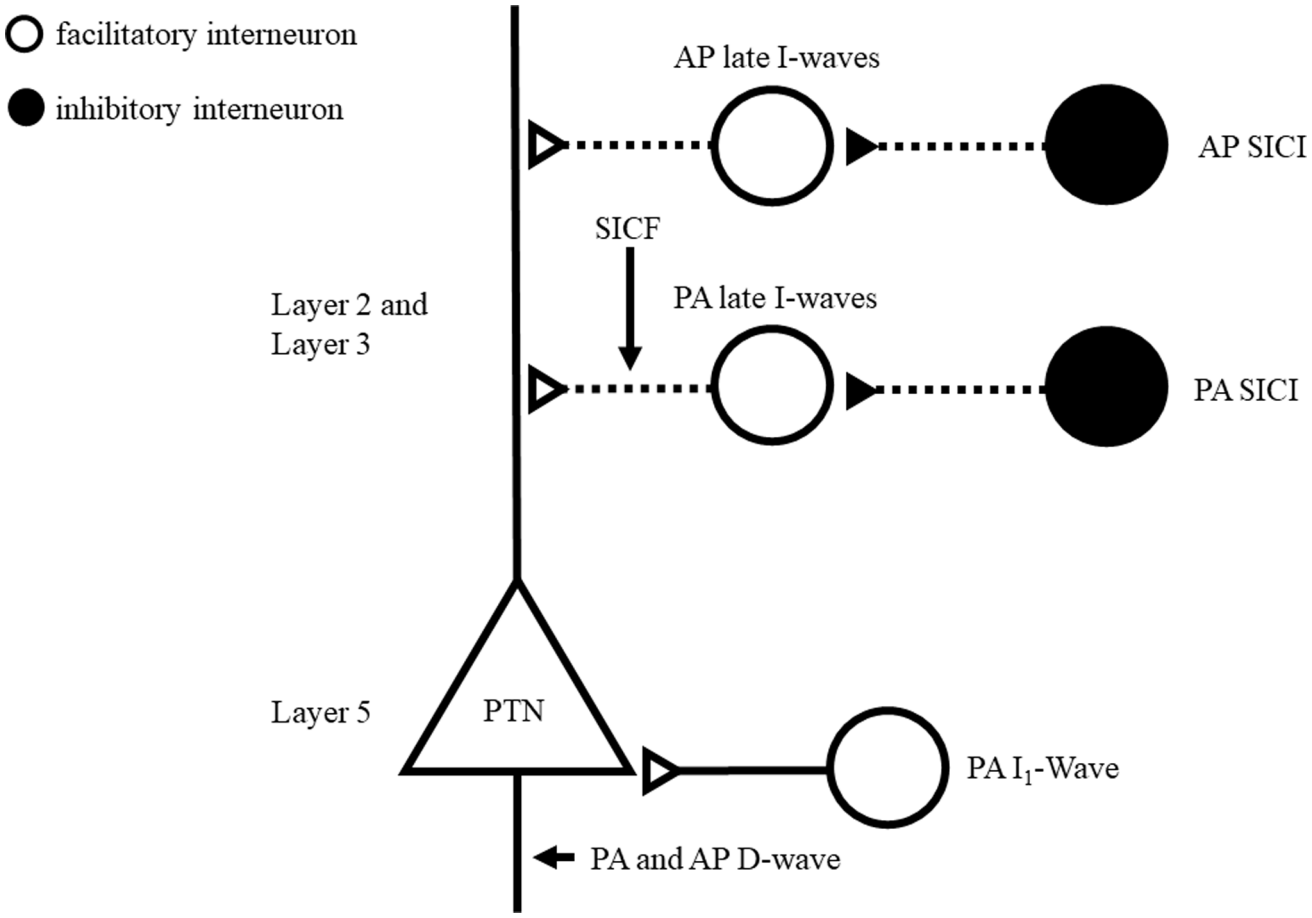
Schematic representation of motor cortex circuits of low back muscles and possible preferential site activation using directional TMS. This model is inspired by Di Lazzaro and Rothwell (2014), Fong et al. (2021), Ziemann (2020). Layers 2 (L2) and 3 (L3) contains interneurons projecting to the pyramidal neurons apical dendrites and layer 5 (L5) includes pyramidal neurons. Two circuits are proposed for PA-TMS late I-wave and AP-TMS delayed late I-wave. GABAergic neurons recruited by SICI paradigms are suggested to connect to corresponding circuit. It remains unclear if different TMS directions recruit different GABAergic SICI circuits. SICF originates through direct excitation (second stimulus) of the axon of interneurons of the late I-wave pathway, which were made hyper excitable by the first stimulus and is a non-synaptic mechanism. Open circles indicate excitatory neurons; Filled circles indicate inhibitory neurons. TMS, transcranial magnetic stimulation; PA, posteroanterior, AP, anteroposterior SICI, short interval intracortical inhibition.

Intracortical inhibitory and facilitatory circuits can be probed with paired-pulse TMS protocols and have been well documented in hand muscles (Reis et al., 2008). Similar TMS protocols have been applied while testing M1 representation of low back muscles (Massé-Alarie et al., 2016b; Elgueta-Cancino et al., 2019; Massé-Alarie et al., 2022). To better understand the neural circuits underlying TMS outcomes, pharmacological agents and epidural recording have been combined to TMS (Ziemann, 2015; Di Lazzaro et al., 2018). However, these techniques have not been applied (pharmaco-TMS) or are not possible to use (epidural recording) in TMS studies targeting low back muscles. Considering that there are similarities in response to paired-pulse TMS of the hand and back muscles, we assume that similar mechanisms are at play. Nonetheless, the lack of pharmacological and epidural recording studies needs to be considered while reading the next sections. Figure 3 schematized potential circuits influencing the corticospinal cells projecting to low back muscles.

#### 4.2.1. Cortical excitability of intracortical inhibitory circuits

Gamma-aminobutyric acid (GABA) is the main inhibitory neurotransmitter in the central nervous system (Chebib and Johnston, 1999). It binds to two main classes of receptors: GABA_A_ and GABA_B_ which mediate fast inhibition and slow prolonged inhibition, respectively (Chebib and Johnston, 1999). For TMS hand studies, SICI and cortical SP would reflect the inhibitory level of neuronal circuits mediated by GABA_A_ and GABA_B_, respectively (Reis et al., 2008). Results from this review suggest similar mechanisms for low back muscles. Indeed, SICI was consistently obtained with AP-TMS (Goss et al., 2011; Massé-Alarie et al., 2016b; Elgueta-Cancino et al., 2019; Desmons et al., 2021) and PA-TMS (Massé-Alarie et al., 2016a; Chiou et al., 2018a,b, 2020;Desmons et al., 2021; Shraim et al., 2022). AP-TMS has been shown to produce more SICI than PA-TMS (Desmons et al., 2021) in line with TMS hand studies (Cirillo and Byblow, 2016; Sale et al., 2016). Direct activation of axons of pyramidal cells is achieved by PA-TMS at high intensity and elicits the earliest volleys composing the MEP termed D(direct)-wave. D-wave is followed by early then late I (indirect)-waves (Ziemann, 2020). I-waves are suggested to originate at the cortical level through synaptic input from interneuronal circuitries connecting onto corticomotoneuronal cells as depicted in Figure 3 (Ziemann, 2020). At motor threshold intensities, PA-TMS recruits only early and late I-waves while AP-TMS recruits preferentially late I-waves (Ziemann, 2020). SICI affects later I-waves but depending if PA- or AP-TMS is used, different late I-waves circuits are suggested to be at play as depicted in Figure 3 (Di Lazzaro et al., 2012; Fong et al., 2021). This is supported by larger inhibition elicited by AP-TMS compared to PA-TMS of low back muscles (Desmons et al., 2021).

The contralateral SP describes the relative electromyographic silence observed following the MEP (Ziemann, 2015). Contralateral SP duration (≈46 ms) in low back (Strutton et al., 2004; Massé-Alarie et al., 2016a, 2017) is shorter than in hand muscles (≈100–350 ms) (Fuhr et al., 1991; Inghilleri et al., 1993; Chen et al., 1999b). Late part (>50 ms) of SP is suggested to be mediated by intracortical mechanisms (GABA_B_) (Siebner et al., 1998; Stetkarova and Kofler, 2013) while the early part (0–50 ms) is thought to originate from the spinal level (Fuhr et al., 1991). It is unclear why the duration of the silent period is shorter for low back muscles. It may results from less cortical neurons projecting to low back muscles compared to hand (Ferbert et al., 1992), hence, a smaller cortical contribution to SP in low back muscles, although this needs to be further studied. Testing long-interval intracortical inhibition, that is GABA_B_-mediated (Reis et al., 2008), in low back muscles could help to resolve this question.

S/LAI combine a peripheral and a cortical stimulation to investigate sensorimotor interactions (Reis et al., 2008). For SAI (19–50 ms), it is suggested that the inhibition is GABA_A_-mediated within M1 (Tokimura et al., 2000) and associated with cholinergic function (Turco et al., 2018). In contrast, LAI (200–1,000 ms) neural circuits remain unclear, but its late onset suggests the contribution of GABA_B_ circuits (Chen et al., 1999a; Turco et al., 2018). Only one study tested the effect of various peripheral conditioning stimulations (cutaneous, muscle) at multiple intervals on corticospinal excitability. A significant inhibition was only presents at ISI 60 ms following muscle stimulation, suggesting no consistent peripheral modulation on M1 excitability (Massé-Alarie et al., 2022).

#### 4.2.2. Cortical excitability of intracortical facilitatory circuits

Intracortical facilitation (ICF) and short intracortical facilitation (SICF) protocols reflect the excitability of excitatory circuits within M1 (Reis et al., 2008).

Using ICF protocol for low back muscles did not result in consistent facilitation with PA-TMS (Desmons et al., 2021; Shraim et al., 2022). However, facilitation with AP-TMS was reported (Goss et al., 2011), but the intensity of stimulation was not based on the motor threshold and may explain that other studies did not observe such facilitation (Massé-Alarie et al., 2016b; Desmons et al., 2021). For hand muscles, ICF can only be elicited by PA-TMS (Reis et al., 2008). Similarly, ICF is absent during voluntary contraction of a hand muscle (Ridding et al., 1995). This might explain why significant ICF has been rarely reported for back muscles, given that these muscles are generally tested using muscle contraction (Ferbert et al., 1992; O’Connell et al., 2007). No TMS study was able to identify the mechanism underlying ICF (Ziemann et al., 1996; Reis et al., 2008). For SICF, significant facilitation was frequently observed in low back muscle using different parameters (Massé-Alarie et al., 2016a, 2016b, 2017; Shraim et al., 2022). SICF originates through direct excitation (second stimulus) of the axon of interneurons of the late I-wave pathway, which were made hyper excitable by the first stimulus and is a non-synaptic mechanism (Figure 3) (Ziemann, 2020). SICF appears to be the only tested protocol able to probe intracortical excitatory in low back muscles.

### 4.3. Testing inter-hemispheric and regional influences on M1

**Figure 4:**
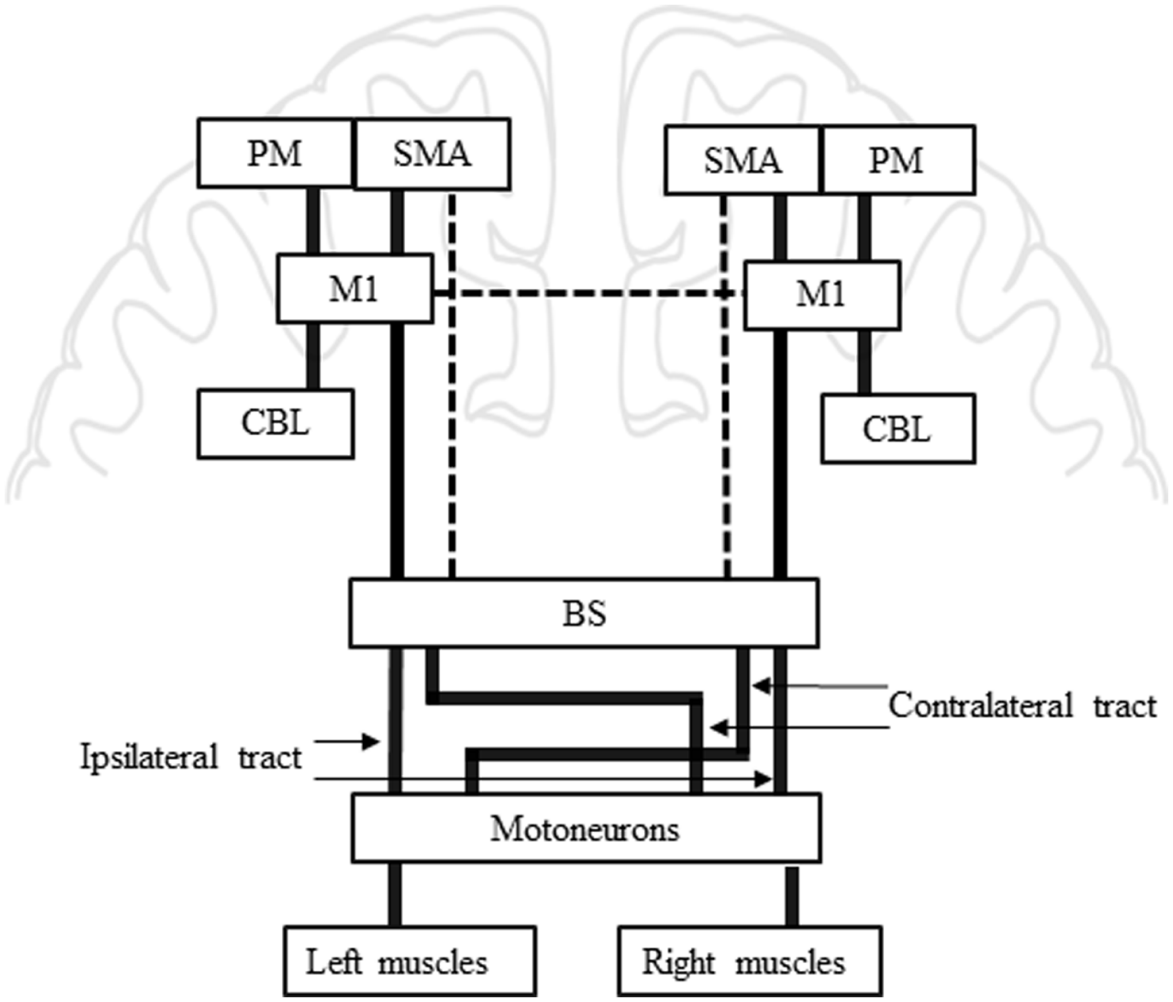
Summary of potential inter-regional influences on M1 for low back muscles. This model is inspired by Reis et al., (2008); Takakusaki (2017). Bold lines from PM, SMA and CBL toward M1: PM, SMA and CBL are known to influence M1 during motor preparation and execution in limb muscles, similar mechanisms may be at play in low back muscles control. Bold lines from M1 toward BS: M1 is usually known as the centre of movement execution, pyramidal cells in M1 are the origin of the descending corticospinal tract. Bold lines between BS and Motoneurons: The pyramidal tract crosses the midline at the level of the BS to reach the muscle contralateral to the stimulated hemisphere. Ipsilateral MEP in low back muscles suggest an ipsilateral tract from one hemisphere to the corresponding ipsilateral muscle. The ipsilateral tract does not cross in the BS. Dashed line from SMA toward BS: SMA has been suggested to be involved in execution of postural command and the strong bilateral projections observed in non-human primates suggest a projection toward motoneurons. Dashed line between both M1s: Interhemispheric influence is suggested between both M1 for low back muscle. BS, brainstem; CBL, cerebellum; M1, primary motor cortex; MEP, motor evoked potential; PM, premotor; SMA, supplementary motor area.

Figure 4 proposes potential interhemispheric (between M1s) and interregional (e.g., between premotor and M1) influences on M1 that potentially contribute to the control of low back muscles based on postural control literature (Massion, 1992; Coffman et al., 2011; Takakusaki, 2017) although most have yet to be formally established in the control of low back muscles. For low back muscles, IHI was tested in only one study and a trend for a modulation of the MEP test was found at 6-ms ISI (Jean-Charles et al., 2017). Cortical (e.g., SMA and PM) or subcortical areas (cerebellum) have not been investigated in low back muscles although they are critical in postural control (Figure 4) (Massion, 1992; Coffman et al., 2011; Takakusaki, 2017). The involvement of SMA, PM and cerebellum have been successfully tested with dual coil protocol for hand muscles [for review see: (Van Malderen et al., 2022)]. Therefore, a specific involvement could also be expected in low back muscles. For example, strong inputs from M1 and SMA trunk representation toward the cerebellum have been shown in non-human primates and are suggested to influence descending control involved in the regulation of posture (Coffman et al., 2011). In addition, reticulospinal (Galea et al., 2010), vestibulospinal and propriospinal pathways (Pierrotdeseilligny, 1996) have been proposed to contribute to postural control (Ferbert et al., 1992; Chiou et al., 2016; Jean-Charles et al., 2017) and to communicate with cortical motor areas (Davey et al., 2002; Takakusaki, 2017). Neurophysiological techniques such as vestibular stimulation (Ali et al., 2003; Guillaud et al., 2020; Desgagnes et al., 2021), startle reflex (Carlsen and Maslovat, 2019) and stretch reflex (Skotte et al., 2005; Rohel et al., 2022) that could indirectly probe subcortical structures (e.g., brainstem circuits) could be combined with TMS in future studies. Overall, Figure 4 highlights the importance of continuing to investigate the role and interactions of neural regions in the control of low back muscles since almost no study has been done in this area yet.

### 4.4. Methodological considerations

Low back muscles responses to TMS were investigated in only 44 studies in healthy participants, compared to the thousands of studies published for hand muscles. It is obvious that methodological (Rossini et al., 2015) and security (Rossi et al., 2009, 2011, 2021) guidelines and checklists (Chipchase et al., 2012; Pellegrini et al., 2020) developed for TMS are not optimised to study low back muscles.

Sixteen of the included studies do not use a MT and 17 do not stimulate over the hotspot as recommended by (Groppa et al., 2012), which limits the interpretation of their results. This is explained by the difficulty to elicit MEPs of low back muscles. Indeed, activation of low back muscles are usually required (Ferbert et al., 1992) in addition to the use of a coil producing a stronger magnetic field than the standard figure-of-eight coil. For example, most studies using a figure-of-eight stimulated at 100% MSO (Tsao et al., 2011c; Burns et al., 2017; Elgueta-Cancino et al., 2019). The use of a fixed stimulation intensity unrelated to MT precludes between-group comparisons and does not allow to test most paired-pulse protocols. Figure-of-eight coil is mainly used for mapping purpose due to its focality (Tsao et al., 2011a; Schabrun et al., 2017) and the possibility to remain tangential to every point of the skull. In contrast, the double cone coil is less focal but allow to obtain a MT for low back muscles (Davey et al., 2002; Chiou et al., 2020; Sasaki et al., 2020). Its shape does not fit with all skull architectures that complexifies mapping more lateral and anterior cortical areas (Desmons et al., 2021). The availability of a stronger figure-of-eight coil (e.g., D70^2^ - The Magstim Co., Whitland, United Kingdom) allows a good trade-off between focality and power that needs to be considered especially for mapping studies of low back muscles (Massé-Alarie et al., 2022; Shraim et al., 2022). Considering that most protocols necessitate the use of a MT to set TMS parameters (e.g., CS and TS intensities), the double cone or D70^2^ coils should be preferred.

To define a MT for active hand muscles, a cut-off value of 200 μV peak-to-peak MEP amplitude is usually recommended (Groppa et al., 2012). However, MEPs of axial muscles are often of small absolute amplitudes and with large variability between participants that may be due, *inter alia*, to subcutaneous fat or electrode placement. Thus, using a cut-off value is inappropriate, and MEP visual identification is often preferred by researchers (Dishman et al., 2008).

Finally, most included studies were realised on young adults (average age: 28.21(3.46) years old), hence, little is known about the effect of age on MEP outcomes of low back muscles (Pellegrini et al., 2020). Future studies should consider testing the effect of age and sex on TMS variables to better understand their impact on corticomotor control of low back muscles.

## 5. Conclusion

This systematic review of the literature reports results from TMS studies testing corticomotor control of low back muscles in healthy humans. Current TMS studies suggest bilateral projections from one M1 to low back muscles on both sides, although the nature of these projections remain to be determined. Also, current findings suggest the presence of intracortical inhibitory and excitatory circuits in M1 involved in the control of low back muscles. Functional cortico-cortical interactions remain to be investigated in future studies. In addition, some methodological factors must be more controlled (hotspot, MT, coil selection) in future TMS studies of low back muscles to improve the interpretation of results. The current findings are of fundamental interest for improving the understanding of corticomotor control of low back muscles before investigating clinical populations (e.g., low back pain, stroke).

## Author contributions

MD and H-MA designed the systematic review and determined the research question. MD prepared the research strategies and complete the search of the articles. MD and MT extracted the data, performed the methodological quality assessment and prepared the tables. MD and H-MA wrote the manuscript and prepared the figures. MD, MT, CM and H-MA approved the final version of the manuscript submitted for publication. All authors contributed to the article and approved the submitted version.

## Funding

This work was funded by a Discovery grant from the Natural Sciences and Engineering Research Council of Canada (RGPIN-2019-06529). H-MA and CM are supported by a research scholar from Fonds de recherche du Québec – Santé (respectively #281961 and 251649). MD is supported by scholarship from Fonds de recherche du Québec – Santé (289953) and Cirris (Year 2019). The funder had no role in study design, data collection and analysis, decision to publish, or preparation of the manuscript.

## Conflict of interest

The authors declare that the research was conducted in the absence of any commercial or financial relationships that could be construed as a potential conflict of interest.

## Publisher’s note

All claims expressed in this article are solely those of the authors and do not necessarily represent those of their affiliated organizations, or those of the publisher, the editors and the reviewers. Any product that may be evaluated in this article, or claim that may be made by its manufacturer, is not guaranteed or endorsed by the publisher.
