# Supplementary material for: Contribution of neural circuits tested by transcranial magnetic stimulation in corticomotor control of low back muscle: a systematic review
Source: Front Neurosci. 2023 May 25;17:1180816. doi: 10.3389/fnins.2023.1180816 (PMC10247989; doi:10.3389/fnins.2023.1180816)
Supplement: Supplementary file 1 [file Table_1.DOCX]

| **Supplementary material 1.** Research strategies. | |
| --- | --- |
| Database | CINAHL |
| Research steps | Research strategies |
| 1 | ((neuro N1 (modulat* OR stimul*)) OR neurostimul* OR (cortical N2 excitab*) OR (brain N2 (mapping OR map OR mapped)) OR (stimul* N3 ((transcranial OR (Trans N1 cranial) OR magnetic* OR transmagnetic*) OR ((single OR paired) N2 pulse))) OR (stimul* N2 (magnetique or transcranien* or trans cranien*))) |
| 2 | ((motor N2 (evoked OR potenti?l*)) OR (evoked N2 potenti?l* N2 (somatosensory or somato sensory)) OR (moteur* N2 (potenti?l* OR evoqu*))) |
| 3 | (cervicomedullary or (cervico N1 medullary)) |
| 4 | ((transcranial OR (trans N1 cranial) OR stimul*) N2 (electri*)) |
| 5 | ((stimul* N2 (vestibul* OR galvanic)) OR (vestibular N2 (evoked OR potentials)) OR (gvs and stimul*)) |
| 6 | (((reflex* or reaction*1 OR re?ponse*) N1 (stretch* OR etirement* OR tendineu* OR myotati* OR osteotendineu* OR tendon*)) OR (muscle N3 tap) OR (tendon* N1 jerk)) |
| 7 | (((reflex* OR reaction*1 OR re?ponse*) N1 (withdrawal* OR retrait OR (flexion spinal defense) OR flexor OR (flexor N1 withdrawal*) OR nocicepti* OR (pathologi* N1 shortening)))) |
| 8 | (((reflex* or reaction or reactions OR re?ponse*) N1 (startl* OR moro OR sursaut OR tresaillement OR embrassement OR bras en croix OR righting)) OR ((acoustic OR auditory) N2 stimul*) OR (auditory N2 (potenti?l* OR evoked))) |
| 9 | (h-reflex OR hreflex OR ((reflex* or reaction*1 OR re?ponse*) N1 H) OR ((reflex* or reaction or reactions OR re?ponse*) N1 (cutaneomuscular OR (cutaneo N2 muscular))) OR (stimul* N1 ((nerve or electric)))) |
| 10 | 1 OR 2 OR 3 OR 4 OR 5 OR 6 OR 7 OR 8 OR 9 |
| 11 | MH (Transcranial Magnetic Stimulation OR Electric Stimulation OR Spinal Cord Stimulation OR Electrical Stimulation, Neuromuscular OR Electrical Stimulation, Functional OR Deep Brain Stimulation OR Acoustic Stimulation OR Electrodes Implanted OR Evoked Potentials, Motor OR Evoked Potentials OR  Reflex, Stretch OR Reflex, Monosynaptic OR H-Reflex OR Brain Mapping) |
| 12 | 10 OR 11 |
| 13 | ((low N1 back) OR (muscl* N1 (back or dos or dorsa* or paravertebra* OR para vertebra* OR para spina* OR paraspina* or multifid* or trunk or tronc)) OR multifid* OR erector spinae) |
| 14 | MH (Lumbar Vertebrae OR Back) |
| 15 | 13 OR 14 |
| 16 | 12 AND 15 |
| Database | EMBASE |
| Research steps | Research strategies |
| 1 | ((neuro NEAR/1 (modulat* OR stimul*)) OR neurostimul* OR (cortical NEAR/2 excitab*) OR (brain NEAR/2 (mapping OR map OR mapped)) OR (stimul* NEAR/3 ((transcranial OR (Trans NEAR/1 cranial) OR magnetic* OR transmagnetic*) OR ((single OR paired) NEAR/2 pulse))) OR (stimul* NEAR/2 (magnetique or transcranien* or trans cranien*))) |
| 2 | ((motor NEAR/2 (evoked OR potenti#l*)) OR (evoked NEAR/2 potenti#l* NEAR/2 (somatosensory or somato sensory)) OR (moteur* NEAR/2 (potenti#l* OR evoqu*))) |
| 3 | (cervicomedullary or (cervico NEAR/1 medullary)) |
| 4 | ((transcranial OR (trans NEAR/1 cranial) OR stimul*) NEAR/2 (electri*)) |
| 5 | ((stimul* NEAR/2 (vestibul* OR galvanic)) OR (vestibular NEAR/2 (evoked OR potentials)) OR (gvs and stimul*)) |
| 6 | (((reflex* or reaction*1 OR re?ponse*) NEAR/1 (stretch* OR etirement* OR tendineu* OR myotati* OR osteotendineu* OR tendon*)) OR (muscle NEAR/3 tap) OR (tendon* NEAR/1 jerk)) |
| 7 | (((reflex* OR reaction*1 OR re?ponse*) NEAR/1 (withdrawal* OR retrait OR (flexion spinal defense) OR flexor OR (flexor NEAR/1 withdrawal*) OR nocicepti* OR (pathologi* NEAR/1 shortening)))) |
| 8 | (((reflex* or reaction or reactions OR re?ponse*) NEAR/1 (startl* OR moro OR sursaut OR tresaillement OR embrassement OR bras en croix OR righting)) OR ((acoustic OR auditory) NEAR/2 stimul*) OR (auditory NEAR/2 (potenti#l* OR evoked))) |
| 9 | (h-reflex OR hreflex OR ((reflex* or reaction*1 OR re?ponse*) NEAR/1 H) OR ((reflex* or reaction or reactions OR re?ponse*) NEAR/1 (cuntaneomuscular OR (cutaneo NEAR/2 muscular))) OR (stimul* NEAR/1 ((nerve or electric)))) |
| 10 | 1 OR 2 OR 3 OR 4 OR 5 OR 6 OR 7 OR 8 OR 9 |
| 11 | ‘transcranial magnetic stimulation’/ OR ‘transcranial electrical stimulation’/ OR ‘electrostimulation’/ OR ‘sensory stimulation’/ OR ‘spinal cord stimulation’/ OR ‘motor evoked potential’/ OR ‘vestibular evoked myogenic potential’/ OR ‘cortical excitability’/ OR ‘myotatic reflex’/ OR ‘reflex’/ OR ‘nerve stimulation’/ OR ‘brain depth stimulation’/ OR ‘magnetic stimulation’/ OR ‘nerve cell stimulation’/ OR ‘neuromuscular electrical stimulation’/ OR ‘sacral nerve stimulation’/ OR ‘vagus nerve stimulation’/ OR ‘mechanical stimulation’/ OR ‘thermal stimulation’/ OR ‘auditory stimulation’/ OR ‘nociceptive stimulation’/ OR ‘somatosensory stimulation’/ OR ‘tactile stimulation’/ OR ‘vestibular stimulation’/ OR ‘sacral nerve stimulation’/ OR ‘spinal reflex’/ OR ‘evoked spinal cord response’/ OR ‘sensory evoked potential’/ OR ‘auditory evoked potential’/ OR ‘laser evoked potential’/ OR ‘somatosensory evoked potential’/ OR ‘vestibular evoked potential’/ OR ‘flexor reflex’/ OR ‘Hoffmann reflex’/ OR ‘monosynaptic reflex’/ OR ‘muscle reflex’/ OR ‘myotatic reflex’/ OR ‘polysynaptic reflex’/ OR ‘reflex arc’/ OR ‘righting reflex’/ OR ‘spinal reflex’/ OR ‘startle reflex’/ OR ‘tendon reflex’/ OR ‘withdrawal reflex’/ OR ‘brain mapping’/ |
| 12 | 10 OR 11 |
| 13 | ((low NEAR/1 back) OR (muscl* NEAR/1 (back or dos or dorsa* or paravertebra* OR para vertebra* OR para spina* OR paraspina* or multifid* or trunk or tronc)) OR multifid* OR erector spinae) |
| 14 | ‘back muscle’/ OR ‘erector spinae muscle’/ OR ‘multifidus muscle’/ OR ‘paraspinal muscle’/ OR ‘back’/ OR ‘lumbar region’/ OR ‘lumbosacral region’/ |
| 15 | 13 OR 14 |
| 16 | 12 AND 15 |
| Database | Medline (OVID) |
| Research steps | Research strategies |
| 1 | ((neuro ADJ1 (modulat* OR stimul*)) OR neurostimul* OR (cortical ADJ2 excitab*) OR (brain ADJ2 (mapping OR map OR mapped)) OR (stimul* ADJ3 ((transcranial OR (Trans adj1 cranial) OR magnetic* OR transmagnetic*) OR ((single OR paired) ADJ2 pulse))) OR (stimul* ADJ2 (magnetique or transcranien* or trans cranien*))) |
| 2 | ((motor ADJ2 (evoked OR potenti#l*)) OR (evoked adj2 potenti#l* adj2 (somatosensory or somato sensory)) OR (moteur* ADJ2 (potenti#l* OR evoqu*))) |
| 3 | (cervicomedullary or (cervico adj1 medullary)) |
| 4 | ((transcranial OR (trans adj1 cranial) OR stimul*) ADJ2 (electri*)) |
| 5 | ((stimul* ADJ2 (vestibul* OR galvanic)) OR (vestibular ADJ2 (evoked OR potentials)) OR (gvs and stimul*)) |
| 6 | (((reflex* or reaction*1 OR re?ponse*) adj1 (stretch* OR etirement* OR tendineu* OR myotati* OR osteotendineu* OR tendon*)) OR (muscle ADJ3 tap) OR (tendon* ADJ1 jerk)) |
| 7 | (((reflex* OR reaction*1 OR re?ponse*) adj1 (withdrawal* OR retrait OR (flexion spinal defense) OR flexor OR (flexor adj1 withdrawal*) OR nocicepti* OR (pathologi* adj1 shortening)))) |
| 8 | (((reflex* or reaction or reactions OR re?ponse*) adj1 (startl* OR moro OR sursaut OR tresaillement OR embrassement OR bras en croix OR righting)) OR ((acoustic OR auditory) ADJ2 stimul*) OR (auditory ADJ2 (potenti#l* OR evoked))) |
| 9 | (((reflex* or reaction or reactions OR re?ponse*) adj1 (startl* OR moro OR sursaut OR tresaillement OR embrassement OR bras en croix OR righting)) OR ((acoustic OR auditory) ADJ2 stimul*) OR (auditory ADJ2 (potenti#l* OR evoked))) |
| 10 | 1 OR 2 OR 3 OR 4 OR 5 OR 6 OR 7 OR 8 OR 9 |
| 11 | Transcranial Magnetic Stimulation/ OR Electric Stimulation/ OR Spinal Cord Stimulation/ OR Acoustic Stimulation/ OR Evoked Potentials, Motor/ OR Vestibular Evoked Myogenic Potentials/ OR Evoked Potentials, Auditory/ OR Evoked Potentials/ OR Cortical Excitability/ OR Reflex, Stretch/ OR Reflex, Startle/ OR Reflex, Monosynaptic/ OR H-Reflex/ OR Brain Mapping/ OR Evoked Potentials, Auditory/ OR Reflex, Righting/ OR Evoked Potentials, Somatosensory/ OR Reflex/ |
| 12 | 10 OR 11 |
| 13 | ( (low adj1 back) OR (muscl* adj1 (back or dos or dorsa* or paravertebra* OR para vertebra* OR para spina* OR paraspina* or multifid* or trunk or tronc)) OR (lumbar ADJ1 (spine OR spinal OR vertebral OR fascia)) OR multifid* OR erector spinae) |
| 14 | Back Muscles/ OR Intermediate Back Muscles/ OR Paraspinal Muscles/ OR Superficial Back Muscles/ OR Back/ OR Lumbosacral Region/ |
| 15 | 13 OR 14 |
| 16 | 12 AND 15 |
| Database | Web of Science |
| Research steps | Research strategies |
| 1 | TS=((neuro NEAR/1 (modulat* OR stimul*)) OR neurostimul* OR (cortical NEAR/2 excitab*) OR (brain NEAR/2 (mapping OR map OR mapped)) OR (stimul* NEAR/3 ((transcranial OR (Trans NEAR/1 cranial) OR magnetic* OR transmagnetic*) OR ((single OR paired) NEAR/2 pulse))) OR (stimul* NEAR/2 (magnetique or transcranien* or trans cranien*))) |
| 2 | TS= ((motor NEAR/2 (evoked OR potenti?l*)) OR (evoked NEAR/2 potenti?l* NEAR/2 (somatosensory or somato sensory)) OR (moteur* NEAR/2 (potenti?l* OR evoqu*))) |
| 3 | TS= (cervicomedullary or (cervico NEAR/1 medullary)) |
| 4 | TS= ((transcranial OR (trans NEAR/1 cranial) OR stimul*) NEAR/2 (electri*)) |
| 5 | TS= ((stimul* NEAR/2 (vestibul* OR galvanic)) OR (vestibular NEAR/2 (evoked OR potentials)) OR (gvs and stimul*)) |
| 6 | TS= (((reflex* or reaction*1 OR re?ponse*) NEAR/1 (stretch* OR etirement* OR tendineu* OR myotati* OR osteotendineu* OR tendon*)) OR (muscle NEAR/3 tap) OR (tendon* NEAR/1 jerk)) |
| 7 | TS= (((reflex* OR reaction*1 OR re?ponse*) NEAR/1 (withdrawal* OR retrait OR (flexion spinal defense) OR flexor OR (flexor NEAR/1 withdrawal*) OR nocicepti* OR (pathologi* NEAR/1 shortening)))) |
| 8 | TS= (((reflex* or reaction or reactions OR re?ponse*) NEAR/1 (startl* OR moro OR sursaut OR tresaillement OR embrassement OR bras en croix OR righting)) OR ((acoustic OR auditory) NEAR/2 stimul*) OR (auditory NEAR/2 (potenti?l* OR evoked))) |
| 9 | TS= (h-reflex OR hreflex OR ((reflex* or reaction*1 OR re?ponse*) NEAR/1 H) OR ((reflex* or reaction or reactions OR re?ponse*) NEAR/1 (cuntaneomuscular OR (cutaneo NEAR/2 muscular))) OR (stimul* NEAR/1 ((nerve or electric)))) |
| 10 | 1 OR 2 OR 3 OR 4 OR 5 OR 6 OR 7 OR 8 OR 9 |
| 11 | TS= ((low NEAR/1 back) OR (muscl* NEAR/1 (back or dos or dorsa* or paravertebra* OR para vertebra* OR para spina* OR paraspina* or multifid* or trunk or tronc)) OR multifid* OR erector spinae) |
| 12 | 10 AND 11 |
