# Supplementary material for: Contribution of neural circuits tested by transcranial magnetic stimulation in corticomotor control of low back muscle: a systematic review
Source: Front Neurosci. 2023 May 25;17:1180816. doi: 10.3389/fnins.2023.1180816 (PMC10247989; doi:10.3389/fnins.2023.1180816)
Supplement: Supplementary file 2 [file Table_2.DOCX]

| **Supplementary material.2** Critical appraisal. |
| --- |
| **Chipchase report list- Transcranial Magnetic Stimulation** |
| *Participant factors* |
| **1-Age of subjects:**  Reported: The age of the participants is reported  Controlled: If there are more than one group of participants the age of participants has been statistically compared between groups and is not significantly different.  If there is only one group of participants, the controlled part of this criterion is not applicable (NA). |
| **2-Gender:**  Reported: The gender of the participants is reported  Controlled: If there are more than one group of participants the proportion of male and female has been statistically compared between groups and is not significantly different.  If there is only one group of participants, the controlled part of this criterion is NA |
| **3-Handedness:**  Reported: The handedness of participants is reported.  Controlled: If there is more than one group of participants the proportion of right-handed and left-handed has been statistically compared between groups and is not significantly different.  If there is only one group of participants, the controlled part of this criterion is NA |
| **4-Subjects prescribed medication**  Reported: NA  Controlled: A statement that participants with prescribed medication are excluded is written. (Done if it is written, criteria of exclusion to TMS). |
| **5-Use of CNS active drugs (e.g. anti-convulsant)**  Reported: The use of CNS active drugs of participants is reported. (if no use: NA)  Controlled: NA if no drugs were used to induced specific neuro modulation, if yes properties, dosage and design of utilisation of the drug. |
| **6-Presence of a neurological condition or pain during the experiment**  Reported: NA  Controlled: A statement that participants with (neurological condition or neuroactive drugs) and (those with pain or musculoskeletal disorder) are excluded is written. |
| 7-Any medical conditions  Reported: NA  Controlled: A statement that participants with (medical conditions) are excluded is written. |
| **8-History of specific repetitive motor activity**  Reported: The proportion of participants with a significant history of repetitive motor activity is reported (for instance: musicians, athlete, video games, repetitive work).  Controlled: If there is more than one group of participants the proportion of participants performing repetitive motor activity has been statistically compared between groups and is not significantly different.  If there is only one group of participants, the controlled part of this criterion is NA |
| *Methodological factors* |
| **9-Position of contact of EMG electrodes**  Reported: The preparation procedure of EMG electrodes placement is described with sufficient details in order to allow replication. For intrinsic hand muscle, a statement that the electrodes were placed in a belly-tendon or belly-belly configuration is sufficient. For the other muscles, the exact configuration is reported. The citation of a recognised standard is sufficient.  Controlled: Citations are provided in order justify the EMG preparation method (SENIAM, ISEK, etc.). |
| **10-Amount of relaxation / contraction of target muscles**  Reported: For experiments performed at rest, a method for ensuring that the participant is relaxed (e.g. visual EMG inspection, online EMG feedback) has been reported. For experiments performed during voluntary contraction, target EMG level (e.g. %MVC) is described and visual feedback is given to the participant to achieve such contraction level.  Controlled: The amount of EMG activity immediately before TMS stimulation (e.g. RMS values) has been statistically compared between conditions and is not significantly different. |
| **11-Prior motor activity of muscle to be tested**  Reported: NA  Controlled: The level of motor activities performed between trials (for instance during breaks in the experiments) is controlled in a sound way. This should include at least the instructions about the level of activity wanted (most of the time “stay relaxed”) during the experimental session. For experiments including the evaluation of both active and resting MEPs, the interval between and/or the order of the conditions should be reported and justified to avoid contamination of resting MEPs by prior active contraction. |
| **12-Level of relaxation of muscles other than those being tested**  Reported: NA  Controlled: The process of exclusion of trials based on the amount of relaxation / contraction of other muscles is reported and sound. For instance, visual inspection of pre-stimulus EMG in muscles other than those being tested. |
| **13-Coil type**  Reported: The coil geometry is reported with sufficient details to allow replication  Controlled: Not applicable unless the study has been performed in multiple centers. For multicenter studies, the same coil type has been used for all subjects. |
| **14- Coil orientation / (criteria 14-15 merged)**  Reported: The coil orientation is reported with sufficient details to allow replication.  Controlled: Use of a neuronavigation system in order to control the coil stability. |
| **15-Direction of induced current in the brain**  Reported: The direction of induced current in the brain is reported with sufficient details to allow replication.  Controlled: Use of a neuronavigation system in order to control the coil stability. |
| **16- Coil location and stability (with or without a neuron avigation system)**  Reported: A sound method for ensuring coil stability has been used with or without the use of a neuronavigation system. For instance, marking the stimulation location on participants’s scalp is sufficient.  Controlled: Use of a neuronavigation system in order to control the coil stability. |
| **17-Type of stimulator used (brand)**  Reported: The type of stimulator used is reported.  Controlled: NA unless the study have been performed in multiple centers. For multicenter studies, the same stimulator type / pulse shape have been used for all subjects. |
| **18-Stimulation intensity**  Reported: The stimulation intensity used is reported with sufficient details to allow replication.  Controlled: The intensity of stimulation used is determined individually for each subject (e.g. based on motor threshold or on a standardized targeted MEP size). |
| **19-Pulse shape (monophasic or biphasic)**  Reported: The type of pulse shape used is reported.  Controlled: NA unless the study have been performed in multiple centers. For multicenter studies, the same stimulator type / pulse shape have been used for all subjects. |
| **20-Determination of optimal hotspot**  Reported: The method of determination of optimal hotspot is described with sufficient detail to allow replication or is referenced  Controlled: Appropriate references (or justification with pilot data) are provided to justify hotspot determination method. If more than one muscle is evaluated, the hotspot has to be determined individually for each of the tested muscle. |
| **21-Time between MEP trials**  Reported: The time between MEP trials is reported with sufficient details to allow replication.  Controlled: Time between MEP trials should vary slightly from one trial to the other in order to prevent anticipation of TMS |
| **22-Time between days of testing**  Reported: The time between days of testing is reported with sufficient details to allow replication.  Controlled: If there is more than one group of participants, the time between days of testing should be the same between groups.  If there is only one group of participants, the controlled criterion is NA. If there is only one day of testing reported and controlled criteria are NA. |
| **23-Subject attention (arousal) during testing**  Reported: NA  Controlled: A sound method is presented in order to control subjects’ attention during testing and reported with sufficient detail to allow replication. A task controlling attention (for instance: counting stimuli, detecting visual stimuli, pain rating after each block, etc.) should be used when testing subjects at rest. When active TMS is evaluated, the presence of a target contraction to sustain with feedback is sufficient to control subjects’ attention |
| **24-Method to determine motor threshold**  Reported: Presence of a statement explaining how motor threshold have been determined with sufficient detail to allow replication or referenced.  Controlled: Appropriate references are provided to justify motor threshold determination method. If more than one muscle is evaluated, the motor threshold has to be determined individually for each of the tested muscle. |
| **25-Number of MEP measures made**  Reported: The number of MEP measure in each condition is reported.  Controlled: The number of MEP included in the analyses and the way excluded stimulations were managed is described. For instance, if x stimulation were not valid, were they retested or simply excluded from the analyses? |
| **26-Paired pulse only: Intensity of test pulse**  Reported: The stimulation intensity used for test pulse is reported with sufficient details to allow replication.  Controlled: The intensity of stimulation used is determined individually for each subject (e.g. based on motor threshold or on a standardized targeted MEP size). |
| **27-Paired pulse only: Intensity of conditioning pulse**  Reported: The stimulation intensity used for conditioning pulse is reported with sufficient details to allow replication.  Controlled: The intensity of stimulation used is determined individually for each subject (e.g. based on motor threshold or on a standardized targeted MEP size). |
| **28-Paired pulse only: Inter-stimulus interval**  Reported: The stimulation inter-stimulus interval is reported with sufficient details to allow replication.  Controlled: NA |
| *Analytical factors* |
| **29-Method for determining MEP size during analysis**  Reported: The method used to quantify MEP amplitude is described with sufficient detail to allow replication  Controlled: If the method is reported it is automatically controlled |
| **30-Size of unconditioned MEP**  Reported: The amplitude of unconditioned MEP is reported.  Controlled: NA |
