# Supplementary material for: Contribution of neural circuits tested by transcranial magnetic stimulation in corticomotor control of low back muscle: a systematic review
Source: Front Neurosci. 2023 May 25;17:1180816. doi: 10.3389/fnins.2023.1180816 (PMC10247989; doi:10.3389/fnins.2023.1180816)
Supplement: Supplementary file 3 [file Table_3.DOCX]

| **Supplementary material 3.** Characteristics of the population in included studies of the systematic review. | | | | | |
| --- | --- | --- | --- | --- | --- |
| Study | Groups (n) | Age | % Female | Height | Handedness (% right) |
| Behrendt 2016 | 14 | 24.5 | 21 |  |  |
| Burns 2017 | 11 | 27(5) | 45 |  | 100 |
| Cariga 2002 | 10 | 40 | 50 |  |  |
| Chiou 2016 | 20 | 22(3) | 50 | 174(9) |  |
| Chiou 2018a | 34 | 25(6) | 41 | 175(11) |  |
| Chiou 2018b | 16 | 29.7(10.9) | 50 |  | 94 |
| Chiou 2020 | 30 | 21(3) | 50 |  | 20 |
| Clark 2011 | 10 | 22.9(1.9) | 50 | 174.5(8.4) |  |
| Davey 2002 | 10 | 24.2(2.5) | 40 |  | 100 |
| Davey 2004 | 3 | 36.6(8.4) | 67 |  |  |
| Desmons 2021 | 12 | 27(6.6) | 58 |  | 92 |
| Dishman 2008 | 23 | 20-40 (min, max) | 39 |  |  |
| Elgueta-Cancino 2019 | 12 | 27(8) | 33 | 173(6) | 100 |
| Ertekin 1998 | 34 | 20-67 (min, max) | 29 |  |  |
| Ferbert 1992 | 9 | 25-38 (min, max) |  |  |  |
| Fujiwara 2001 | 11 | 28-62 (min, max) | 18 |  |  |
| Fujiwara 2009 | 9 | 22-33 (min, max) | 78 |  |  |
| Fulton 2002 | 6 | 22.18(0.26) |  | 178(1.7) |  |
| Goss 2011 | 17 | 24.6(5.3) | 12 | 178.1(8.5) |  |
| Hashimoto 2000 | 7 | 22-29 (min, max) | 0 |  |  |
| Jaberzadeh 2013 | 7 | 38.3(7.3) | 57 |  | 100 |
| Jean-Charles 2017 | 25 | 28.0 (6.7) | 52 |  | 100 |
| Jiang 2021 | 9 | 24.6(1.8) | 50 | 174.8(5.2) | 100 |
| Kuppuswamy 2008 | 18 | 38(15) | 33 |  |  |
| Lehner 2017 | 35 | 24(3) | 51 |  | 100 |
| Li 2021 | 12 | 28.2(4.0) | 50 | 167.3(8.1) | 100 |
| Massé-Alarie 2016a | 13 | 37.6(12.5) | 54 | 166.9(10.0) | 100 |
| Massé-Alarie 2016b | 10 | 26(9) | 40 | 171(8) | 100 |
| Massé-Alarie 2018 | 15 | 26(5) | 40 |  | 93 |
| Massé-Alarie 2022 | 14 | 27(6) | 57 | 166(9) | 93 |
| Nowicky 2001 | 14 | 21-52 (min, max) | 43 |  | 93 |
| Oconnell 2007 | 12 | 25 (Mean) 19-51(min, max) | 83 |  | 83 |
| Rowland 2021 | 20 | 20.3(1.4) |  | 171.5(7.0) | 95 |
| Sasaki 2020 | 12 | 25.9(3.3) |  | 173.3(5.3) |  |
| Sasaki 2021 | 12 | 24.8(1.5) |  | 173.5(5.3) | 100 |
| Schabrun 2018 | 10 | 27(6) | 40 |  | 100 |
| Shraim 2022 | 28 | 23.6(4.6) | 47 | 173(7.2) | 84 |
| Stalder 1995 | 14 | 29,4 (Mean) 24-38 (min ,max) | 50 |  |  |
| Strutton 2005 | 11 | 35.9 (3.2) | 36 |  |  |
| Taniguchi 1999 | 15 | 30 (Mean) 24-47 (min, max) | 0 |  |  |
| Tsao 2011a | 11 | 24(5) | 55 | 170(8) | 100 |
| Tsao 2011b | 11 | 24(5) | 55 | 170(8) | 100 |
| Tsao 2011c | 9 | 25(4) | 44 | 176(10) |  |
| Urban 1994 | 30 | 48.1(15.8) | 30 |  |  |
