# Supplementary material for: Contribution of neural circuits tested by transcranial magnetic stimulation in corticomotor control of low back muscle: a systematic review
Source: Front Neurosci. 2023 May 25;17:1180816. doi: 10.3389/fnins.2023.1180816 (PMC10247989; doi:10.3389/fnins.2023.1180816)
Supplement: Supplementary file 4 [file Table_4.DOCX]

| **Supplementary material 4.** Methodology of included studies. | | | | | | | | |
| --- | --- | --- | --- | --- | --- | --- | --- | --- |
| Study | Position of sensors | | | EMG recording | | Type of coil/ pulse shape/ stimulator brand | Position of the participants | Chipchase’s score (% score) |
| Behrendt 2016 | L1 | | | sEMG | | Figure of eight coil type D-B80 - biphasic - R30 MagPro | Seated in an upright position | 60 |
| Burns 2017 | L3 and L5 – bilaterally – spacing 2.0 cm - 3 cm from spinous processes | | | sEMG | | Figure of eight coil (70mm Ø)– Magstim 200^2^ | Sitting forward with the back straight | 68.6 |
| Cariga 2002 | T12 – bilaterally – spacing 3 cm - 1 cm from spinous processes | | | sEMG | | Double cone coil | Sitting on a chair | 34.3 |
| Chiou 2016 | | L4 – bilaterally – spacing 2cm – 3cm from spinous processes | | sEMG | Figure of eight (100mm Ø) - monophasic – Magstim 200^2^ | | Stood upright | 71.4 |
| Chiou 2018a | T12 – bilaterally – spacing 2cm – 3 cm from spinous processes | | | sEMG | | Figure of eight coil (100 mm Ø) - round coil (90mm Ø) - monophasic – Magstim 200^2^ | Stood or lying | 65.8 |
| Chiou 2018b | T12 - bilaterally | | | sEMG | | Double cone coil (110 mm Ø) – round coil (90mm Ø) - bat-wing coil (90 mm Ø) - monophasic – Magstim 200^2^ | Seated | 70.7 |
| Chiou 2020 | T12 – bilaterally – spacing 3 cm from spinous processes | | | sEMG | | Figure of eight coil (100 mm Ø) - Double cone coil (110 mm Ø)) – monophasic - Magstim 200^2^ | Seated | 61.7 |
| Clark 2011 | L2 – bilaterally – spacing 6 cm – 2-3cm from spinous processes | | | sEMG | | Custom-modified double cone coil (110 mm Ø) – Magstim 200^2^ | Seated | 45.7 |
| Davey 2002 | T12 – bilaterally – spacing 4 cm – 3 and 7 cm from spinous processes | | | sEMG | | Angled double cone coil– Magstim 200^2^ | Standing or lying | 60 |
| Davey 2004 | T12 – bilaterally | | | sEMG | | Angled double cone coil – Magstim 200^2^ | Stood up-micro gravity | 40 |
| Desmons 2021 | L3-L4 – right side | | | sEMG | | Angled double cone coil (126mm Ø) – Figure of eight (70mm Ø) – monophasic - Magstim 200^2^ | Seated | 87.8 |
| Dishman 2008 | L5-S1 – right side | | | sEMG | | Angled double cone coil (125mm Ø) – Magstim 200^2^ | Lying | 40 |
| Elgueta-Cancino 2019 | L2 and L5– right side – fwe adjacent spinous process – sEMG 3 cm from spinous processes (L2) – sEMG 1 cm from spinous processes (L5) | | | fwe – sEMG | | Double cone coil (70 mm Ø) – monophasic – Magstim Bistim^2^ | Prone | 56.1 |
| Ertekin 1998 | T12 and L3 – right side – spacing 3.2 cm -2 cm from spinous processes – T12 and L3 needle electromyogram | | | sEMG | | Round coil (90mm Ø) – monophasic – Magstim 200^2^ | Prone, sitting and standing | 31.4 |
| Ferbert 1992 | L3-L4 – bilaterally - spacing 2.5-3 cm – 3.5 cm from spinous processes | | | sEMG - fwe | | Figure of eight coil (90 mm and 50 mm Ø– Magstim 200^2^ | Seated and bent slightly forward | 31.4 |
| Fujiwara 2001 | L3-L4 – bilaterally - 2 cm spacing – 3.5 cm from spinous processes | | | sEMG | | Figure of eight coil – Dantec MagLite | Seated on a semi-reclining chair | 45.7 |
| Fujiwara 2009 | Right side | | | sEMG | | Figure of eight coil (70 mm Ø) - Double cone coil (70 mm Ø)– Magstim 200^2^ | Seated | 45.7 |
| Fulton 2002 | L3 - L4 - bilaterally – spacing 2 cm – 3 cm from spinous processes | | | sEMG | | Angled double cone figure of eight coil (125 mm Ø) –Magstim 200^2^ | Prone | 40 |
| Goss 2011 | L2-L4 – bilaterally – spacing 6 cm | | | sEMG | | Double cone coil (110 mm Ø) –Magstim 200^2^ | Seated | 51.2 |
| Hashimoto 2000 | T12 (sEMG) and T12 intramuscular EMG (iEMG)– 1-2 cm from spinous process (iEMG) | | | sEMG - fwe | | Plane figure of eight coil (100 mm Ø) – Magnetic stimulator (Nihon Kohden) | Prone | 32.4 |
| Jaberzadeh 2013 | L2-L3 – right side, 5 cm from spinous processes | | | sEMG | | Flat figure of eight coil (200 mm Ø)– Magstim 200^2^ | Sitting supported in a semi-reclined chair | 65.7 |
| Jean-Charles 2017 | L1 – bilaterally – 2cm from spinous process | | | sEMG | | Figure of eight coil (70mm Ø) – circular coil (90mm Ø) -monophasic –MagStim 200^2^ | Seated back unsupported while maintaining a straight back | 90.2 |
| Jiang et al., 2021 | L2-L4 – bilaterally | | | hdEMG 3×15 array (45 electrodes) | | Figure of eight coil (70mm Ø) - biphasic - Magstim Rapid^2^ | Seated | 57.1 |
| Kuppuswamy 2008 | | | L4 – bilaterally – 3 and 6cm from spinous process | sEMG | Angled double cone coil (110mm Ø) – figure of eight coil (70mm) – MagStim 200^2^ | | Seated | 51.4 |
| Lehner 2017 | | | L3 – bilaterally – 3.3cm from spinous process | sEMG | Figure of eight coil (70mm Ø) –monophasic – MagStim 200^2^ | | Seated | 77.1 |
| Li et al., 2021 | | | L5 – bilaterally | sEMG | Figure of eight (70mm Ø) – Rui Chi magnetic stimulator | | Seated | 76.3 |
| Massé-Alarie 2016a | | | L5 – bilaterally – 2-3cm from spinous process | sEMG | Double cone coil (70mm Ø) – monophasic – MagStim 200^2^ | | Seated and lean slightly forward with the lumbar spine in lordosis | 73.2 |
| Massé-Alarie 2016b | | | L4-L5 – right side | fwe | Double cone coil (70mm Ø)) –monophasic – MagStim 200^2^ | | Prone | 70.7 |
| Massé-Alarie 2018 | | | L5 – right side – 2-3cm from spinous process | sEMG | Custom double cone coil (70mm Ø) –monophasic – Magstim Bistim | | Seated and maintain the lumbar spine in lordosis | 96.8 |
| Massé-Alarie 2022 | T12 and L5 - right side – 4-5 cm from spinous process | | | sEMG | | D70^2^ coil (70mm Ø) – monophasic - Magstim  Bistim^2^ | Seated | 90.2 |
| Nowicky 2001 | L3 – bilaterally – 3cm from spinous process | | | sEMG | | Double cone coil (125mm Ø) – MagStim 200^2^ | Prone | 51.4 |
| O’Connell 2007 | L3-L5 – bilaterally – 3cm from L3 spinous process and 1 cm from L5 spinous process | | | sEMG | | Figure of eight coil (70mm Ø)– biphasic – Magstim Rapid Family | Seated forward in a chair and maintaining extension of the back | 60 |
| Rowland 2021 | T12 – bilaterally -3cm from spinous process | | | sEMG | | D70 Alpha coil (70mm Ø) – MagStim 200^2^ | Seated | 59.4 |
| Sasaki 2020 | T12 – bilaterally -3cm from spinous process | | | sEMG | | Angled double cone coil (110mm Ø) –monophasic – MagStim 200^2^ | Seated | 62.2 |
| Sasaki 2021 | T12 – right side -3cm from spinous process | | | sEMG | | Angled double cone coil (110mm Ø) –monophasic – MagStim 200^2^ | Seated | 80 |
| Schabrun 2018 | L3 – 3cm from spinous process | | | sEMG | | Figure of eight coil– MagStim 200^2^ | Seated | 66.7 |
| Shraim 2022 | L3 and L5 – right side - 3cm from L3 spinous process and 1 cm from L5 spinous process | | | sEMG | | Double cone coil (70mm Ø)) –monophasic – MagStim 2002  D70^2^ coil (70mm Ø) – monophasic - Magstim  Bistim2 | Seated | 92.7 |
| Stalder 1995 | L3-L4 – 2-3cm medially from spinous process | | | sEMG | | Circular coil (90mm Ø) – Custom stimulator | Lying | 40 |
| Strutton 2005 | L4 – bilaterally – 3 and 6 cm from spinous process | | | sEMG | | Angled double cone coil – MagStim 200^2^ | Prone | 45.7 |
| Taniguchi 1999 | T12 to L5 – right side – 3.5cm from interspinous spaces | | | sEMG | | Circular coil (90mm Ø) – Mag Lite | Prone | 34.3 |
| Tsao 2011a | L1-L4 – bilaterally inserted electrodes adjacent to L1 and L4 spinous process – right side sEMG 5cm from spinous process of L4 and 3cm from spinous process of L1 | | | sEMG - fwe | | Figure of eight coil (70mm Ø) – MagStim 200^2^ | Seated and leaning forward with the back straight | 68.6 |
| Tsao 2011b | L4 – bilaterally | | | fwe | | Figure of eight coil (70mm Ø) –monophasic – MagStim 200^2^ | Sitting and leaning forward with the back  straight. | 57.1 |
| Tsao 2011c | L2 – bilaterally – 5cm from spinous process | | | sEMG | | Double cone coil (90mm Ø) –monophasic – MagStim 200^2^ | Prone | 60 |
| Urban 1994 | T12-L1 – bilaterally | | | sEMG | | Circular coil (90mm Ø) – MagStim 200S | Prone | 22.8 |
| Median (min, max) | | | | | | | | 60 (22.8,96.8) |
| *fwe: fine wire electrodes; hdEMG: electromyography high density sEMG: surface electromyography.* | | | | | | | | |
