# Supplementary material for: Contribution of neural circuits tested by transcranial magnetic stimulation in corticomotor control of low back muscle: a systematic review
Source: Front Neurosci. 2023 May 25;17:1180816. doi: 10.3389/fnins.2023.1180816 (PMC10247989; doi:10.3389/fnins.2023.1180816)
Supplement: Supplementary file 5 [file Table_5.DOCX]

| **Supplementary material 5.** Frequency distribution of the TMS checklist ratings | | |
| --- | --- | --- |
| Factors (adapted from Chipchase et al., 2012) | Reported (n/T.A.) | Controlled (n/T.A.) |
| **Participant factors** |  |  |
| Age of subjects | 42/44 (95.5) | NA |
| Gender of subjects | 40/44 (90.9) | NA |
| Handedness | 23/44 (52.3) | NA |
| Subjects prescribed medication | NA | 22/44 (50.0) |
| Use of CNS active drugs (e.g. anti-convulsant) | NA | NA |
| Presence of neurological condition / psychiatric disorders | NA | 31/44 (70.5) |
| Any medical conditions | NA | 42/44 (95.5) |
| History of specific repetitive motor activity | 3/44 (6.8) | NA |
| **Methodological factors** |  |  |
| Position and contact of EMG electrodes | 36/44 (81.8) | 13/44 (29.5) |
| Amount of relaxation/ contraction of target muscles | 27/44 (61.4) | 15/44 (34.1) |
| Prior motor activity of muscle to be tested | NA | 22/44 (50.0) |
| Level of relaxation of muscles other than those being tested | NA | 1/44 (2.3) |
| Coil type (size and geometry) | 44/44 (100.0) | NA |
| Coil orientation | 38/44 (86.4) | 7/44 (15.9) |
| Direction of induced current in the brain | 37/44 (84.1) | 7/44 (15.9) |
| Coil location and stability (with or without neuronav) | 28/44 (63.6) | 7/44 (15.9) |
| Type of stimulator used (brand) | 42/44 (95.5) | NA |
| Stimulation intensity | 41/44 (93.2) | 27/44 (61.4) |
| Pulse shape (monophasic or biphasic) | 22/44 (50.0) | NA |
| Determination of optimal hotspot | 20/44 (45.5) | 7/44 (15.9) |
| Time between MEP trials | 23/44 (52.3) | 18/44 (40.9) |
| Time between days of testing | 3/3 (100.0) | NA |
| Subject attention (level of arousal) during testing | NA | 32/44 (72.7) |
| Method of determining threshold (active/resting) | 27/44 (61.4) | 9/44 (20.5) |
| Number of MEP measures made | 42/44 (95.5) | 16/44 (36.4) |
| Paired pulse only: Intensity of test pulse | 11/11 (100.0) | 11/11 (100.0) |
| Paired pulse only: Intensity of conditioning pulse | 11/11 (100.0) | 11/11 (100.0) |
| Paired pulse only: Interstimulus interval | 11/11 (100.0) | NA |
| **Analytical factors** |  |  |
| Method for determining MEP size during analysis | 17/44 (43.2) | 18/44 (40.9) |
| Size of unconditioned MEP | 11/11 (100.0) | NA |
