# Supplementary material for: Contribution of neural circuits tested by transcranial magnetic stimulation in corticomotor control of low back muscle: a systematic review
Source: Front Neurosci. 2023 May 25;17:1180816. doi: 10.3389/fnins.2023.1180816 (PMC10247989; doi:10.3389/fnins.2023.1180816)
Supplement: Supplementary file 6 [file Table_6.DOCX]

| **Supplementary material 6.** Interrater agreement for the TMS checklist appraisal tool. | | |
| --- | --- | --- |
| Factors (adapted from Chipchase et al., 2012) | Reported  Gwet's AC1 (95% CI) | Controlled  Gwet's AC1 (95% CI) |
| **Participant factors** |  |  |
| Age of subjects | 0.9(0.8,1.0) | 1.0(1.0,1.0) |
| Gender of subjects | 0.9(0.8,1.0) | 1.0(1.0,1.0) |
| Handedness | 0.9(0.8,1.0) | 1.0(1.0,1.0) |
| Subjects prescribed medication | NA | 0.6(0.4,0.9) |
| Use of CNS active drugs (e.g. anti-convulsant) | 1.0(1.0,1.0) | 1.0(1.0,1.0) |
| Presence of neurological condition / psychiatric disorders | NA | 0.7(0.5,0.9) |
| Any medical conditions | NA | 0.5(0.3,0.8) |
| History of specific repetitive motor activity | 0.9(0.8,1.0) | 1.0(0.9,1.0) |
| **Methodological factors** |  |  |
| Position and contact of EMG electrodes | 0.8(0.6,0.9) | 0.7(0.5,0.9) |
| Amount of relaxation/ contraction of target muscles | 0.6(0.3,0.8) | 0.6(0.3,0.8) |
| Prior motor activity of muscle to be tested | NA | 0.4(0.1,0.7) |
| Level of relaxation of muscles other than those being tested | NA | 0.9(0.9,1.0) |
| Coil type (size and geometry) | 0.9(0.8,1.0) | 1.0(1.0-1.0) |
| Coil orientation | 0.8(0.7,1.0) | 0.9(0.8,1.0) |
| Direction of induced current in the brain | 0.8(0.6,1.0) | 0.9(0.8,1.0) |
| Coil location and stability (with or without neuronavigation) | 0.6(0.3,0.8) | 0.9(0.8,1.0) |
| Type of stimulator used (brand) | 0.9(0.8,1.0) | 0.9(0.9,1.0) |
| Stimulation intensity | 0.6(0.4,0.9) | 0.8(0.6,1.0) |
| Pulse shape (monophasic or biphasic) | 0.1(-0.2,0.4) | 1.0(1.0,1.0) |
| Determination of optimal hotspot | 0.6(0.3,0.8) | 0.9(0.7,1.0) |
| Time between MEP trials | 0.5(0.2,0.8) | 0.8(0.7,1.0) |
| Time between days of testing | -1.0(-1.0,-1.0) | 1.0(0.9,1.0) |
| Subject attention (level of arousal) during testing | NA | 0.8(0.6,1.0) |
| Method of determining threshold (active/resting) | 0.9(0.8,1.0) | 0.8(0.6,1.0) |
| Number of MEP measures made | 0.8(0.6,0.9) | 0.4(0.1,0.7) |
| Paired pulse only: Intensity of test pulse | 0.9(0.7,1.0) | 0.9(0.8,1.0) |
| Paired pulse only: Intensity of conditioning pulse | 0.9(0.7,1.0) | 0.9(0.8,1.0) |
| Paired pulse only: Interstimulus interval | 0.9(0.7,1.0) | NA |
| **Analytical factors** |  |  |
| Method for determining MEP size during analysis | 0.7(0.5,0.9) | 0.7(0.5,0.9) |
| Size of unconditioned MEP | -0.5(-1.0,0.0) | NA |
| Median(min; max) | 0.9(-1.0,1.0) | 0.9(0.1,1.0) |
| *CNS: central nervous system; EMG: electromyography; MEP: motor evoked potential; NA: not applicable; N: number of times the factor was rated as ‘reported’ or ‘controlled’; T.A.: total number of times where the factor was applicable across all studies.* | | |
