# Supplementary material for: Contribution of neural circuits tested by transcranial magnetic stimulation in corticomotor control of low back muscle: a systematic review
Source: Front Neurosci. 2023 May 25;17:1180816. doi: 10.3389/fnins.2023.1180816 (PMC10247989; doi:10.3389/fnins.2023.1180816)
Supplement: Supplementary file 7 [file Table_7.DOCX]

| **Supplementary material 7.** Latencies and cortical silent period of included studies. | | | | |
| --- | --- | --- | --- | --- |
| Study | Current direction induced in brain | Stimulation localization – side of MEP recording – neuronavigation | Task, level of activation and TMS intensity | Main results (mean) SD |
| Burns 2017  n=11 | Postero-anterior; | Hotspot – contralateral  matched side for pain free – contralateral muscle – Brainsight neuronavigation; | *Muscle activation:* 20% MVC;  *TMS intensity:* 100% MSO; | *cSP - latency*  81.4(49.3) ms; |
| Cariga 2002  n=10 | Postero-anterior; | Vertex – right and left muscle; | *Muscle activation:* Rest;  *TMS intensity:* Intensity at minimal back muscle motor threshold; | Right:16.1(0.7) ms;  Left: 15.5(0.6) ms; |
| Chiou 2018a  n=34 | Postero-anterior; | Hotspot – most excitable hemisphere – contralateral muscle; | *Task:* during motor preparation and reaction time periods;  *Muscle activation:* 10-20% MVC;  *TMS intensity:* 120% aMT; | *cMEP:*  Standing:13.0(2.7) ms;  Lying: 12.0(2.2) ms; |
| Chiou 2018b  n=16 | Postero-anterior; | Hotspot – right hemisphere – contralateral muscle; | *Muscle activation:* Rest;  *TMS intensity:* minimum to elicit an MEP with a peak-to-peak amplitude of ~0.1 mV (89.7(12.7) % MSO); | *cMEP:*  16.7(1.9) ms; |
| Chiou 2020  n=7 | Postero-anterior; | Hotspot – ipsilateral to the dominant arm – contralateral muscle; | *TMS intensity:* generate MEPs in ES at peak-to-peak amplitudes of ~ 0.1 mV; | *cMEP:*  14.6(2.0)ms; |
| Davey 2002  n=10 | Postero-anterior; | Vertex – right and left muscle; | *TMS intensity:* 105-110% rMT; | Right: 14.8(0.4) ms;  Left: 14.6(0.6) ms; |
| Desmons 2021  n=12 | Postero-anterior (PA) and antero-posterior (AP) | Hotspot – left hemisphere – contralateral muscle – Brainsight neuronavigation | *Muscle activation:* 15 % MVC;  *TMS intensity:* 120–160% aMT; | *cMEP:*  PA: 13.9(2.4) ms;  AP: 14.8(3.1) ms; |
| Ertekin 1998  n=34 | Postero-anterior; | Hotspot – left hemisphere – contralateral muscle; | *Muscle activation:* Slight activity;  *TMS intensity:* aMT: 70 to 100% MSO; | *cMEP - sEMG:*  *Sitting: T12: 15.1(0.9) ms;*  *Prone: T12: 15.4(2.1) ms;*  *Standing: T12 15.2(1.4) ms;*  Sitting: L3: 17.0(0.9) ms;  Prone: L3: 17.2(1.6) ms;  *Standing*: L3 16.9(1.6) ms;  *cMEP - fwe:*  *Standing*: L3 17.3(1.6) ms;  *Erected: T12 16.1(2.0) ms;* |
| Ferbert 1992  n=9 | Postero-anterior; | 0, 2, 4, 6 cm lateral to vertex- 4 cm lateral and 2 cm anterior to vertex – both hemisphere – contralateral muscle; | *Muscle activation:* ES activation;  *TMS intensity:* 100% MSO; | *cMEP:*  MEP: Range: 13-24 ms; |
| Fujiwara 2001  n=6 | Antero-posterior | 20 scalp sites per hemisphere – contralateral and ipsilateral muscle | *TMS intensity:* 100% MSO; | *cMEP:*  15.9(4.9) ms;  *iMEP:*  15.2(2.8) ms; |
| Fujiwara 2009  n=9 | Antero-posterior; | Hotspot – left hemisphere – contralateral muscle; | *Muscle activation:* maintaining neck flexion with chin on and chin off;  *TMS intensity:*120% rMT; | *cMEP:*  Chin-on: 13.7(1.6) ms;  Chin-off: 12.6(1.7) ms; |
| Fulton 2002  n=6 | Postero-anterior; | Vertex – right and left muscle; | *TMS intensity:* before and after rowing exercise/ 120% rMT; | *Pre exercise - non rowers:*  right ES: 16.5(0.9) ms;  left ES: 17.3(1.3) ms;  *Pre exercise - elite rower*  left ES: 14.4(0.9) ms;  right ES: 14.8(1.2)ms; |
| Hashimoto 2000  n=7 |  | 2cm lateral and 2 cm anterior to vertex – contralateral muscle; | *Muscle activation:* slight voluntary contraction;  *TMS intensity:* 60-70%MSO; | *cMEP- fwe:*  13.3(0.5) ms; |
| Jaberzadeh 2013  n=7 | Latero-medial; | Hotspot– left hemisphere – contralateral muscle; | *Muscle activation:* 0%, 25%, 50%, 75% and 100% MVC;  *TMS intensity:* 120% aMT/ aMT: 71±4.7; | *cMEP:*  low back extension: 15.4(0.7) ms;  forced expiration during breath holding: 16.5(0.9) ms; |
| Jean-Charles 2017  n=12 | Postero-anterior; | Hotspot - Right hemisphere – contralateral and ipsilateral muscles – Brainsight neuronavigation; | *Tasks:* maintaining a straight back (shoulder task-ES stabilizer) or isometric extension combined with right inclination trunk (trunk task-ES agonist);  *TMS intensity:* 130% aMT; | *Baseline:*  *iSP:* present in 41.6% of subjects;  *Shoulder task:*  *iMEP:*  14.8(1.4)ms;  *iSP - latency*:  41.8(4.4)ms;  *iSP - duration*:  30.1(2.2)ms; |
| Kuppuswamy 2008  n=18 | Postero-anterior; | Vertex – right and left muscle; | *Muscle activation:* maintain a 90-degree arm abduction left or right against a 2,4,6 or 8N resistance, MEP recorded contralateral or ipsilateral to abducted arm;  *TMS intensity:* 110-120% aMT; | *110% aMT, resistance 8N:*  *MEP contralateral to arm:*  right MEP: 16.3ms;  left MEP: 18.9 ms;  *MEP ipsilateral to arm:*  right MEP: 11.3 ms;  left MEP: 11.8 ms;  *120% aMT, resistance 8N:*  Contralateral: right:17.0(0.8) ms; left:16.0(0.8) ms;  Ipsilateral: right: 13.6(0.4) ms; left: 13.9(1.0) ms;  *120% aMT, all resistances:*  Contralateral: right: 16,6(0.9) ms; left: 16.1(1,0) ms;  Ipsilateral: right: 14,5(0,7) ms; left: 13,7(0,8) ms; |
| Li 2021  n=12 | Postero-anterior; | Hotspot – contralateral muscle; | *Muscle activation:* 11.55% MVC;  *TMS intensity:* 120% rMT; | *cMEP:*  Right hemisphere: 14.5(3.3) ms;  Left hemisphere: 13.4(4.9) ms; |
| Massé-Alarie 2016a  n=13 | Postero-anterior; | Hotspot – both hemispheres – contralateral muscle; | *Muscle activation:* sitting EMG level;  *TMS intensity:* 120% aMT; | *cSP -* *duration*  right: 48.8(32.4) ms;  left: 46.8(22.1) ms; |
| Nowicky 2001  n=14 | Postero-anterior; | Vertex – right and left muscle; | *Task (i):* forced expiration;  *Task (ii):* bilateral trunk extension;  *TMS intensity:* 50-85%MSO rMT: 56.5±7.3%; | *Forced expiration:*  Rest:  right MEP: 18.6(0.6) ms ;  left MEP: 18.2(0.6) ms;  10-20%MVC:  right MEP: 1.0 (0.4) ms;  left MEP: 1.3(0.5) ms*;  *Trunk extension:*  Rest:  right MEP: 18.9(0.8) ms;  left MEP: 18.7(0.8) ms;  10-20%MVC:  right MEP: 1.5(0.5) ms;  left: 1.9(0.6) ms*;  **MEP latency decreased in activity compared to rest* |
| O’Connell 2007  n=10 | Postero-anterior; | Hotspot – both hemispheres – contralateral and ipsilateral muscle; | *Muscle activation:* 30%MVC;  *TMS intensity:* 100%MSO; | *cMEP:*  19.5(4.8) ms;  *iMEP:*  24.2(3.8) ms; |
| Schabrun 2018  n=10 | Postero-anterior; | Vertex – left hemisphere – contralateral muscle – Brainsight neuronavigation; | *Muscle activation:* 20%MVC;  *TMS intensity:* 100%MSO; | *cSP - duration*  87.5(8.3) ms; |
| Stalder 1995  n=14 | Postero-anterior; | Vertex | *Muscle activation:* relax ES;  *TMS intensity:* 80-100%MSO; | 15.2(1.4) ms; |
| Strutton 2005  n=11 | Postero-anterior; | Vertex; | *Muscle activation:* 20%MVC;  *TMS intensity:* 100-120%aMT;  aMT=36±2.11%MSO;  SP threshold: 29.18±1.57%MSO; | 120%aMT: 16.1(0.7) ms;  cSP latency: 38.5(2.0) ms;  cSP duration: 41.8(4.12) ms; |
| Taniguchi 1999  n=15 |  | Vertex; | *Muscle activation:* 20%MVC;  *TMS intensity:* 90-100%MSO; | T12-L1=18.5(2.0) ms;  L1-L2=19.5(2.4) ms;  L2-L3=19.8(1.9) ms;  L3-L4=20.3(1.8) ms;  L4-L5=20.8(1.5) ms; |
| Tsao 2011a  n=10 | Postero-anterior; | Hotspot and other scalp sites on 5x7cm grid system – both hemispheres – contralateral muscle; | *Muscle activation:* 20%MVC;  *TMS intensity:* 100%MSO; | *cMEP - fwe: [L3-L5]*  Left LES-L1:17.9(2.2) ms;  Right LES-L1:17.8(2.5) ms;  Left DM-L4:15.6(1.9) ms;  Right DM-L4:15.2(0.9) ms;  left LES-L4:18.4(2.5) ms;  Right LES-L4:18.3(3.0) ms;  *cMEP - sEMG:*  Right LES-L4 post:15.8(1.3) ant:16.2(0.8) ms; |
| Tsao 2011b  n=11 | Postero-anterior; | Hotspot – contralateral muscle; | *Muscle activation:* 20%MVC;  *TMS intensity:* 100%MSO; | *cMEP:*  Average across sides:  DM:15.8(1.1) ms;  LES:18.0(3.0) ms; |
| Tsao 2011c  n=9 | Postero-anterior; | Hotspot – both hemispheres – contralateral and ipsilateral muscle; | *Muscle activation (i):* rest;  *TMS intensity (i):* 50-90%MSO;  *Muscle activation (ii):* 10%MVC of transversus abdominis;  *TMS intensity (ii):* 30-80%MSO; | *cMEP:*rest: 14.2(1.2) ms;  abdominal contraction: 14.0(0.8)ms;  *iMEP:*rest: 15.7(1.0) ms;  abdominal contraction: 14.9(0.7) ms; |
| Urban 1994  n=30 | Postero-anterior; | Vertex – right and left muscle; | *Muscle activation*: slight activation multifidus;  *TMS intensity:* 75-95%MSO; | Right: 13.2(1.2) ms;  Left: 13.5(1.2) ms; |
| *a/rMT: active/resting motor threshold; DM: deep multifidus; ES: erector spinae; fwe: fine wire electrodes; MSO: maximal stimulator output; sEMG: surface electromyography; c/iSP: contralateral/ipsilateral silent period; c/iMEP: contralateral/ipsilateral MEP; SD: standard deviation.* | | | | |
