# Supplementary material for: Contribution of neural circuits tested by transcranial magnetic stimulation in corticomotor control of low back muscle: a systematic review
Source: Front Neurosci. 2023 May 25;17:1180816. doi: 10.3389/fnins.2023.1180816 (PMC10247989; doi:10.3389/fnins.2023.1180816)
Supplement: Supplementary file 8 [file Table_8.DOCX]

| **Supplementary material 8.** Paired pulse TMS of included studies. | | | | |
| --- | --- | --- | --- | --- |
| Study | Current direction induced in brain | Stimulation localization – neuronavigation | Task -Muscle activation - intensity of stimulation - motor threshold | Results (mean) SD |
| SICI | | | | |
| Chiou 2018a  n=18 | Postero-anterior; | Hotspot – most excitable hemisphere – contralateral muscle; | *Task:* during motor preparation period and baseline of shoulder flexion task;  *TMS intensity:* SICI (CS 70% aMT; TS 120% aMT; ISI 2.5ms); | 57.4(19.4)% MEP test; |
| Chiou 2018b  n=8 | Postero-anterior; | Hotspot – right hemisphere – contralateral muscle; | *Task:* elbow flexion and index finger abduction;  *Muscle activation:*20% MVC;  *TMS intensity:* SICI (CS: 70% aMT; TS: set at an intensity needed to elicit an MEP with a peak-to-peak amplitude of ~0.1 mV (86.4±15.2% MSO); ISI: 2.5ms); | SICI decreased during elbow flexion and index finger abduction compared with rest; |
| Chiou 2020  n=7 | Postero-anterior; | Hotspot – ipsilateral to the dominant arm – contralateral muscle; | *TMS intensity:* SICI (CS 70% AMT; TS: intensity to evoke MEP of ~ 0.1 mV; ISI: 2.5ms); | 52.9(6.5)% MEP test; |
| Goss 2011  n=12 | Antero-posterior; | Vertex; | In two sessions with 30min to 1h between;  *TMS intensity:* SICI (CS: 40 or 50% MSO; TS: 80 or 90% MSO; ISI: 3ms); | *session 1:*  67.9(18.9) % MEP test;  *session 2:*  73.2(22.6) % MEP test; |
| Desmons 2021  n=12 | Postero-anterior (PA-TMS) and antero-posterior (AP-TMS); | Hotspot – left hemisphere - contralateral muscle – Brainsight neuronavigation; | *Muscle activation:* 15% MVC;  *TMS intensity:* SICI (CS 80% aMT TS 120% aMT; ISI: 2 and 3ms); | *PA-TMS:*  2ms: -4.2(27.2) % MEP test;  3ms: -14.7(27.9) % MEP test;  *AP-TMS:*  2ms: -32.4 (34.1) % MEP test;  3ms: -38.0(29.5) % MEP test |
| Elgueta-Cancino 2019  n=10 | Antero-posterior; | Hotspot – left hemisphere contralateral muscle; | *Muscle activation:* 10-20% MVC;  *TMS intensity:* SICI (CS: 70% aMT; TS: 120-159% aMT; ISI 2 and 3ms); | 2ms: 82.0 % MEP test;  3ms: 57.3 % MEP test; |
| Massé-Alarie 2016a  n=13 | Postero-anterior; | Hotspot – both hemisphere – contralateral muscle; | *TMS intensity:* SICI (CS: 70% aMT; TS: 120% aMT; ISI: 2 ms);  aMT: left M1: 50.9 (8.7); right M1: 50.6(8.0); | left M1: 77.3(25.5) % MEP test;  right M1: 90.1(29.4) % MEP test; |
| Massé-Alarie 2016b  n=10 | Antero-posterior; | Hotspot – left hemisphere – contralateral muscle; | *Muscle activation:* 10-20%MVC;  *TMS intensity*: CS: 70-80% aMT; TS: 120-160% aMT; ISI: 2,3,4 and 5 ms;  aMT: 43.5(6.9) % MSO; | 1ms: 57.3(13.8) % MEP test;  2ms: 76.3(28.0) % MEP test;  3ms: 63.9(26.7) % MEP test;  4ms: 68.6(21.6) % MEP test;  5ms: 74.1(23.6) % MEP test; |
| Shraim 2022  n=16 | Postero-anterior; | Hotspot – left hemisphere – contralateral muscle – Brainsight neuronavigation; | *TMS intensity:* CS: 70% aMT; TS: 120% aMT; ISI: 3ms;  aMT: figure of eight: 57.5(10.4); double cone: 40.3(9.1) % MSO; | SICI present in 88% of participant; |
| ICF | | | | |
| Goss 2011  n=12 | Antero-posterior; | Vertex; | In two sessions with 30min to 1h between;  *TMS intensity:* CS: 40 or 50% MSO; TS: 80 or 90% MSO; ISI: 15ms; | *session 1:*  134.1(16.4) % MEP test;  *session 2:*  127.0(27.9) % MEP test; |
| Desmons 2021  n=12 | Postero-anterior (PA-TMS) and antero-posterior (AP-TMS); | Hotspot – left hemisphere – contralateral muscle – Brainsight neuronavigation; | *Muscle activation:* 15% MVC;  *TMS intensity:* CS 80% aMT TS 120% aMT; ISI: 10 and 15ms; | *PA-TMS:*  10ms: -1.5(35.4) % MEP test;  15ms: 13.2(38.6) % MEP test;  *AP-TMS:*  10ms: -1.2(28.2) % MEP test;  15ms: 13.2(43.1) % MEP test; |
| Elgueta-Cancino 2019  n=10 | Antero-posterior; | Hotspot – left hemisphere contralateral muscle; | *Muscle activation:* 10-20% MVC;  *TMS intensity:* CS 80% aMT; TS: 120-159% aMT; ISI 15 ms; | 15ms: 90.6 % MEP test; |
| Massé-Alarie 2016b  n=10 | Antero-posterior; | Hotspot – left hemisphere – contralateral muscle; | *Muscle activation:* 10-20%MVC;  *TMS intensity:* CS: 70-80% aMT; TS: 120-160% aMT; ISI: 10,11,12 and 15 ms;  aMT: 43.5(6.9) % MS); | 10ms: 88.3(51.9) % MEP test;  11ms: 82.9(35.4) % MEP test;  12ms: 75.9(37.8) % MEP test;  15ms: 89.0(39.7) % MEP test; |
| Shraim 2022  n=16 | Postero-anterior; | Hotspot – left hemisphere – contralateral muscle – Brainsight neuronavigation; | *TMS intensity:* CS: 90% aMT; TS: 120% aMT; ISI: 15 ms;  aMT: figure of eight: 57.5(10.4);  double cone: 40.3(9.1) % MSO; | ICF present in 44% of participant; |
| SICF | | | | |
| Massé-Alarie 2016a  n=13 | Postero-anterior; | Hotspot – both hemisphere – contralateral muscle; | *TMS intensity:* CS: 90% aMT; TS: 100% aMT; ISI: 1 ms;  aMT: left M1: 50.9 (8.7);  right M1: 50.6(8.0); | left M1: 186.8(77.9) % MEP test;  right M1: 204.2(51.6) % MEP test; |
| Massé-Alarie 2016b  n=10 | Antero-posterior; | Hotspot – left hemisphere – contralateral muscle; | *Muscle activation:* 10-20%MVC;  *TMS intensity:* CS: 90% aMT; TS: 100% aMT; ISI: 1 ms;  aMT: 43.5(6.9) % MSO; | 161.30(85.2) % MEP test; |
| Shraim 2022  n=16 | Postero-anterior; | Hotspot – left hemisphere – contralateral muscle – Brainsight neuronavigation; | *TMS intensity:* CS: 120% aMT; TS: 90% aMT; ISI: 1.5 ms;  aMT: figure of eight: 57.5(10.4); double cone: 40.3(9.1) % MSO; | SICF present in 44% of participant; |
| IHI | | | | |
| Jean-Charles 2017  n=7 | TS: Postero-anterior 45 degree medial,  CS: Latero-medial; | CS: Hotspot – right hemisphere; TS: Hotspot – left hemisphere – Brainsight neuronavigation; | *Tasks:* unilateral flexion of the right shoulder;  *Muscle activation:* 10%MVC;  *TMS intensity:* Dual coil paradigm (CS right hemisphere 130% aMT; TS left hemisphere:120% aMT; ISI: 2,4,6,8,10,12 and 40ms);  aMT: 49.2±3.8 % MSO; | 6ms: 76.4(9.2) % MEP test;  Not significant for the other ISIs |
| Afferent conditioning stimulation | | | | |
| Massé-Alarie 2022  n=14 | Postero-anterior; | CS: peripheral stimulation in the lumbosacral area (cutaneous noxious, cutaneous non-noxious, muscle stimulation);  TS: hotspot – left hemisphere – Brainsight neuronavigation system;  contralateral muscle; | *Muscle activation:* 15% MVC;  *Stimulation intensity:* CS:  (i) cutaneous noxious: electrical stimulation of the sacrum eliciting pain rated at 3/10;  (ii) cutaneous non-noxious: electrical stimulation of the sacrum at 3 times over perceptual threshold;  (ii) muscle: magnetic stimulation at 110% motor threshold producing a  motor response in superficial lumbar multifidi;  TS: hotspot - 120-130% aMT  ISI : 20, 25, 30, 35, 40, 50, 60, 80, 100 and 200 ms; | *(i) Noxious conditioning stimulation:*  Varies between  -0.03 and 0.04 MEP/EMG ratio (log) – not significant;  *(ii) Non-noxious conditioning stimulation:*  Varies between  -0.03 and 0.04 MEP/EMG ratio (log) – no significant;  *(iii) Muscle conditioning stimulation:*  varies between  -0.07 and 0.07 – significant at ISI 60ms only; |
| Other paired pulse paradigms | | | | |
| Massé-Alarie 2016b  n=10 | Antero-posterior; | Hotspot – left hemisphere – contralateral muscle; | *Muscle activation:* 10-20% MVC;  *TMS intensity:* CS: 70-80% aMT; TS: 120-160% aMT; ISI: 6,7,8 and 9 ms;  aMT: 43.5(6.9) % MSO; | 6ms: 78.9(33.0) % MEP test;  7ms: 87.4(32.8) % MEP test;  8ms: 75.8(11.9) % MEP test;  9ms: 81.2(20.4) % MEP test; |
| *aMT: active motor threshold; CS: conditioning stimuli; MEP: motor evoked potential; rMT: resting motor threshold; SD: standard deviation; TS: test stimuli;* | | | | |
